# Computational and structural evidence for neurotransmitter-mediated modulation of the oligomeric states of human insulin in storage granules

**DOI:** 10.1074/jbc.M117.775924

**Published:** 2017-03-27

**Authors:** Vladimír Palivec, Cristina M. Viola, Mateusz Kozak, Timothy R. Ganderton, Květoslava Křížková, Johan P. Turkenburg, Petra Halušková, Lenka Žáková, Jiří Jiráček, Pavel Jungwirth, Andrzej M. Brzozowski

**Affiliations:** From the § York Structural Biology Laboratory, Department of Chemistry, University of York, Heslington, York YO10 5DD, United Kingdom and; ‡ Institute of Organic Chemistry and Biochemistry, Academy of Sciences of the Czech Republic, v.v.i., Flemingovo nám 2, 166 10 Prague 6, Czech Republic

**Keywords:** crystal structure, dopamine, pancreatic islet, serotonin, vesicles, insulin

## Abstract

Human insulin is a pivotal protein hormone controlling metabolism, growth, and aging and whose malfunctioning underlies diabetes, some cancers, and neurodegeneration. Despite its central position in human physiology, the *in vivo* oligomeric state and conformation of insulin in its storage granules in the pancreas are not known. In contrast, many *in vitro* structures of hexamers of this hormone are available and fall into three conformational states: T_6_, T_3_R^f^_3_, and R_6_. As there is strong evidence for accumulation of neurotransmitters, such as serotonin and dopamine, in insulin storage granules in pancreatic β-cells, we probed by molecular dynamics (MD) and protein crystallography (PC) if these endogenous ligands affect and stabilize insulin oligomers. Parallel studies independently converged on the observation that serotonin binds well within the insulin hexamer (site I), stabilizing it in the T_3_R_3_ conformation. Both methods indicated serotonin binding on the hexamer surface (site III) as well. MD, but not PC, indicated that dopamine was also a good site III ligand. Some of the PC studies also included arginine, which may be abundant in insulin granules upon processing of pro-insulin, and stable T_3_R_3_ hexamers loaded with both serotonin and arginine were obtained. The MD and PC results were supported further by in solution spectroscopic studies with R-state-specific chromophore. Our results indicate that the T_3_R_3_ oligomer is a plausible insulin pancreatic storage form, resulting from its complex interplay with neurotransmitters, and pro-insulin processing products. These findings may have implications for clinical insulin formulations.

## Introduction

Insulin is one of the key human protein hormones that is responsible for the maintenance of metabolic homeostasis, with an influence on cell proliferation and regulation of aging (1, 2). Defects in insulin bioavailability or impaired insulin receptor signaling lead to different pathological conditions such as diabetes (3–5), cancers (6–8), and Alzheimer's disease (9).

Insulin is a 51-amino acid protein consisting of two disulfide-linked chains (A1-A21, B1-B30) and exerts its functions through binding as a monomer to the (αβ)_2_ heterodimer tyrosine-kinase-type insulin receptor (IR)^5^ (10, 11). Insulin is produced from a single chain pro-insulin and stored in pancreatic β-cells in storage granules (termed large dense core vesicles (LDCVs)) from which it is released into the bloodstream in response to elevated blood glucose levels. The first 3D crystal structure of insulin was described by Hodgkin and coworkers in 1969 (12) in the form of its hexameric assembly (with three and two-fold rotational symmetry (*i.e.* trimer of dimers), obtained in the presence of Zn^2+^ ions. Two Zn^2+^ ions were identified in the hexamer on its 3-fold axis, being coordinated by 3 imidazole side chains of His^B10^. This and subsequent similar findings resulted in a generally accepted paradigm that the hexamer is the storage form of insulin in LDCVs in pancreatic β-cells, whereas upon its release into the bloodstream it dissociates to monomers, which represent the active form of the enzyme (13). The pioneering work of D. C. Hodgkin was followed by 3D descriptions of hundreds of *in vitro* studied insulin hexamers, dimers, and monomers (for reviews, see for example Refs. 14–16). However, the actual *in vivo* storage form of insulin in pancreatic β-cells LDCVs is still not known, and it is extrapolated from its *in vitro* structures. Surprisingly, there are more advances into the very elusive nature of insulin/IR interactions (17, 18) than into the *in vivo* form of this hormone, investigation of which presents formidable experimental challenges.

**Figure 1.**
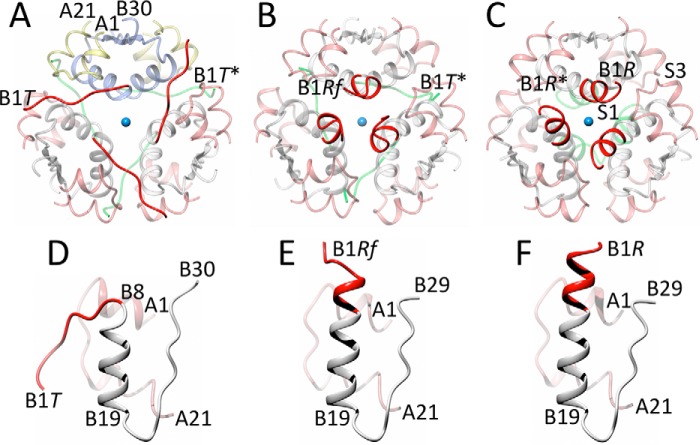
General structural organization of the three main forms of insulin hexamer: T_6_ (*A*), T_3_R^f^_3_ (*B*), and R_6_ (*C*) in the hexamer top view along its 3-fold symmetry axis. Insulin B chains are in *white*, A chains are in *pink*; the chains of one representative insulin dimer are indicated in *A* in *light blue* (B-chains) and *yellow* (A-chains). Some N and C termini of one dimer are also indicated in *A* and only B-chain N termini in *B* and *C*, with * corresponding to the symmetry-related monomer within a dimer. The B1-B8 segments of the B chains that contribute to the largest structural changes in TR transitions are in *red* (*top of the hexamer*) and in *green* (*bottom of the hexamer*). The Zn^2+^ ion is in *blue. D–F*, representative insulin monomers in T, R^f^, and R-state, respectively, with a coloring code as in *A–C*. Typical, main ligand binding sites I and III in the R insulin form are indicated in *C* as S1 and S3 respectively.

The *in vitro* data showed that insulin hexamers can be grouped into three structurally distinct states/families: T_6_, T_3_R^f^_3_, and R_6_ (15, 16), which differ by the conformation of the B1-B6 N termini of the hormone B-chain (see Fig. 1 and supplemental Fig. S1). In the T_6_ hexamers, the initial B1-B6 segments of the B chains are fully extended followed by B7-B10 type II′ β-turns that go into invariant B9-B19 α-helices. In contrast, the B1-B6 segments acquire an α-helical conformation in the R-state, extending the B9-B19 α-helix (19). The insulin R^f^_3_ state is similar to the α-helical R_6_ state; however, the long R-state B-chain helix is shortened to the B3-B19 segment, whereas the B1-B3 residues depart (“fray”) from the α-helical fold (20, 21). The TR^f^ transition can be induced by an increase of, for example, the concentration of Zn^2+^, SCN^−^, and Cl^−^ ions (22) or by the presence of phenolic derivatives (*e.g.* phenol or similar small cyclic alcohols) at low concentrations (23–25). The R^f^R (or full TR) transition can be accomplished by a further increase of the concentration of phenolic ligands (19, 24). The octahedral Zn^2+^ coordination is also a signature of the T-state (assured by three His^B10^ imidazoles and three water molecules), whereas the Zn^2+^ tetrahedral coordination (three His^B10^ and, for example, a Cl^−^ ion) is typical for the R^f^/R-states (*e.g.* Ref. 26). This coordination switch results from a smaller space above the Zn^2+^ ion in the R-state that is obstructed there by a longer B1-B8 helical part of the B9-B19-helix and a newly formed binding pocket for cyclic alcohols on the dimer interface, formed by the side chains of A6–7, A9–11, A16, B7, B11, and B5–7 (the so-called site I, or phenol main-binding site) However, variations of the Zn^2+^ coordination spheres are also observed frequently (27).

Although site I is the main binding cavity for all phenol-like ligands, they have also been identified in other regions of the hexamers; *e.g.* site II, formed by side chains B9, B12, B16, B17, B9, B10, B13; site I/II formed by B10, B14, B16, B17, and B9 (28). Hexamer surface-exposed (between insulin dimers) phenolic site III (A14, A17) has been observed as well (29). The serendipitous character of the discovery of the phenol-stabilized R-state resulted from the bactericide-like applications of phenol in clinical formulations of insulin. Nevertheless, the R-like states of the hormone have clinical importance, as the R^f^/R-trimers/hexamers are more stable (30). This is mostly due to the slower Zn^2+^-solvent exchange, which results in a higher hexamer-monomer dissociation constant. However, the physiological relevance of T/R^f^/R states for an effective insulin/IR binding and for the storage form of the hormone (*e.g.* protection against proteolysis) is still unknown.

Here, we attempt to shed light on a possible conformation of the *in vivo* storage form of insulin in insulin-containing granules in pancreatic β-cells. Although the exact chemical composition of LDCVs still awaits full characterization, they contain Zn^2+^ ions and phenolic neurotransmitters such as dopamine and serotonin, which are involved in regulation of exocytosis of the granules and insulin release (31–33). Therefore, we hypothesize that both neurotransmitters can bind to, and affect the structure of insulin hexamers, assembled in the presence of zinc cations. We probe these effects in parallel via molecular dynamics simulations (MD) and protein X-ray crystallography. These were further supplemented by spectroscopic studies with the use of a sensitive chromophore, 4-hydroxy-3-nitrobenzoic acid (4H3N), which specifically binds to R-state insulin as indicated by a red shift of its absorption spectrum. Moreover, as arginine can accumulate in LDCVs upon processing of pro-insulin at its two Arg-rich sites (34, 35) and as arginine-rich polypeptides (spermine/spermidine) are used in some insulin injections formulations (36, 37), we also probe the structural effect of this amino acid on insulin oligomeric assembly.

Both the MD and X-ray serotonin-related studies point to a stable insulin hexamer-serotonin-site I complex and its dynamic association with site III. Both methodologies excluded dopamine binding in site I. MD simulations also indicate preferential dopamine over serotonin binding to site III, whereas solid-state X-ray analyses yields only serotonin-site III complexes. In addition, crystal structures of serotonin-insulin hexamers loaded with arginine have been obtained that underline a possible more complex, physiological role of insulin-arginine interactions.

Results concerning site I complexes are corroborated by 4H3N spectroscopic experiments, which provide apparent binding constants for interaction of insulin hexamers with phenolic ligands and arginine. Our findings thus provide a novel insight into the storage mechanism of insulin in pancreatic β-cells, also relevant for novel insulin formulations for clinical applications.

## Results

### MD simulations of serotonin, dopamine, and phenol binding in site I

**Figure 2.**
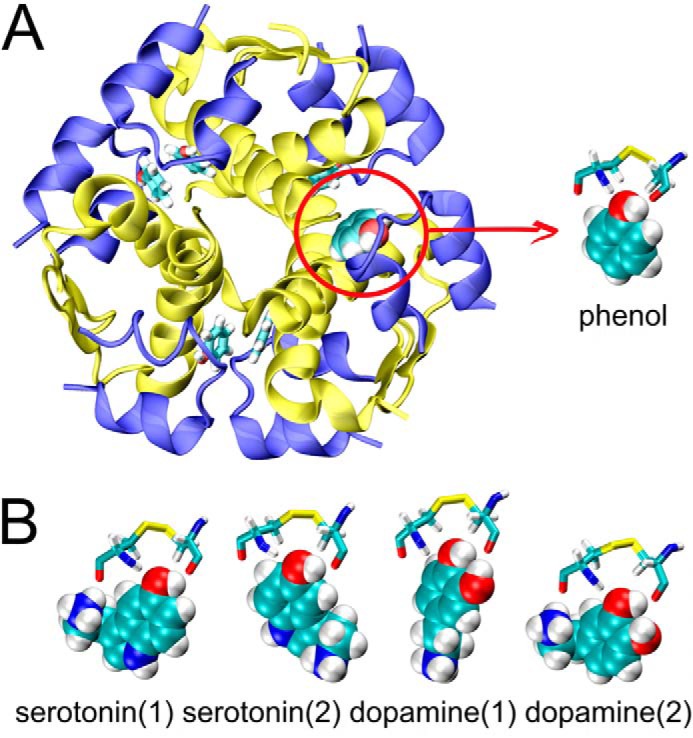
**Starting conformations of phenol (*A*, insulin R_6_ hexamer shown for the sake of clarity of the phenolic pocket location), serotonin (*B*), and dopamine (*B*) molecules (depicted by van der Waals *spheres*) in the phenolic site I pocket (amino acids A6 and A11 involved in the binding are depicted by a *stick model*); two initial orientations of serotonin, and dopamine were considered.**

**Figure 3.**
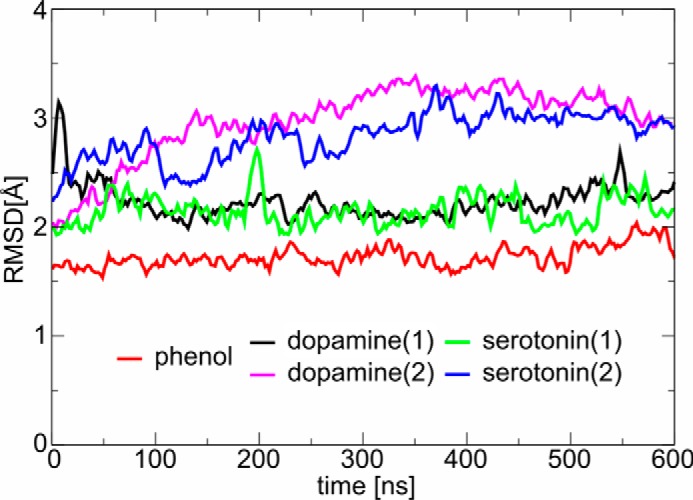
**r.m.s.d. of the protein backbone (heavy atoms) from the insulin R_6_ hexamer NMR structure with different phenolic ligands (phenol, dopamine, or serotonin).** Labels *1* and 2 correspond to the two starting orientations of the neurotransmitter molecules.

Here, we address the question of whether two neurotransmitters, serotonin and dopamine, can substitute phenol in its insulin R_6_ hexamer binding site I. MD simulations were performed for the R_6_ insulin hexamer, where site I-bound phenol was systematically replaced with neurotransmitters (each ligand in two steric-clash-free orientations; see Fig. 2). As expected, the MD simulations yielded the benchmark phenol-bound insulin R_6_-state hexamer (InsPheR_6_) with a low root mean square deviation (r.m.s.d.) of ∼1.6–1.8 Å from the reference InsPheR_6_ structure, indicating that the system does not deviate significantly from the NMR structure during the simulation (Fig. 3).

**Figure 4.**
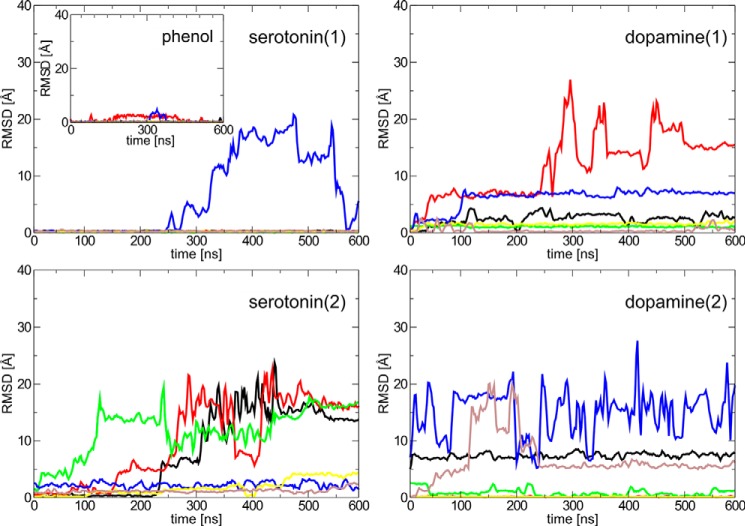
**r.m.s.d. from simulations of all phenolic ligands (serotonin or dopamine in two orientations) from the phenol-binding pockets in the insulin R_6_ hexamers (in total six phenolic ligands per one R_6_ hexamer).** Each line corresponds to the r.m.s.d. of one phenolic ligand from its starting position with respect to the O atom of Cys^A6^ and the N atom of Cys^A11^ involved in a hydrogen bond. A zero or small value of r.m.s.d. means a strong ligand, R_6_ hexamer hydrogen bond, whereas a significant increase of r.m.s.d. indicates breaking of this bond.

Both serotonin (InsSerR_6_) and dopamine (InsDopR_6_) complexes with ligands orientation (**1**) behaved similarly to InsPheR_6_, with the backbone r.m.s.d. just above 2 Å. However, the starting orientation (**2**) of the ligands led to higher r.m.s.d. of ∼2.8 Å (Fig. 2) indicating somewhat larger structural changes. A more direct insight into the behavior of the ligand in site I can be obtained from a direct comparison of their r.m.s.d. (Fig. 4).

The MD simulations confirm that phenol molecules are essentially fixed in site I with a minimal dynamic behavior. Two of the six phenols transiently break HBs (both with Cys^A6^ and Cys^A11^); however, they remain in site I, eventually reestablishing the original geometry of binding. Similarly, all but one of the serotonin molecules in the starting conformation (**1**) stay in site I (only one serotonin ligand temporarily leaves this cavity). However, serotonin in starting geometry (**2**) as well as dopamine in both starting orientations, break most of the crucial hydrogen bonds. Serotonin, but not dopamine, thus essentially mimics phenol interactions observed in InsPheR_6_ with its charged aminoethyl-group-forming HB with backbone carbonyl CO of Cys^A11^, fitting well into the binding site I.

The strength of binding (*i.e.* the binding free energy and the corresponding dissociation constant) of phenol and the two neurotransmitters was calculated using the thermodynamic integration method (see Table 1). The free energy calculations confirmed and quantified the pattern observed in direct MD simulations, indicating a similarly strong binding of phenol and serotonin to site I (*K_d_* of 5.4 × 10^−4^ m and 8.1 × 10^−4^ m, respectively) as well as demonstrating a lack of any stable conformation of dopamine in this cavity (reflected in a positive binding free energy).

**Table 1:** **Standard free energies of binding of phenol, dopamine, and serotonin, molecules to the binding site I (phenolic pocket) together with the corresponding dissociation constants**

Ligand	Δ*G_b_*°	*K_d_*
	*kcal/mol*	[*M*]
Phenol	−4.49 ± 1.55	5.4 × 10^−4^
Dopamine	1.10 ± 1.73	6.3
Serotonin	−4.24 ± 1.87	8.1 × 10^−4^

### MD simulations of serotonin and dopamine probing the hexamer surface sites

**Figure 5.**
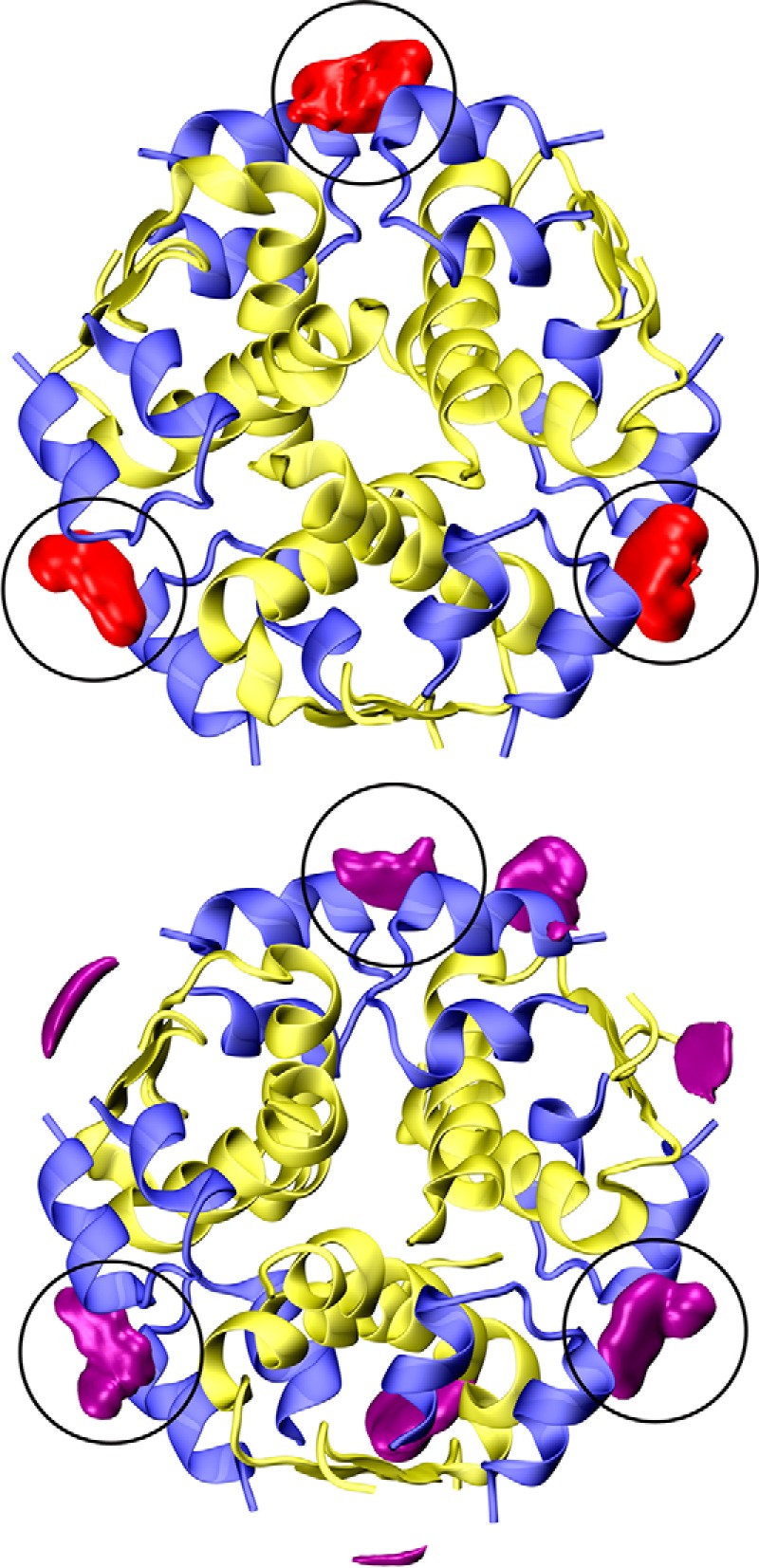
**Dopamine (*top*, *red*) and serotonin (*bottom*, *magenta*) spatial distributions around the insulin R_6_ hexamer (*top view*).** The A/B-chains are in *blue* and *yellow*, respectively; the same isodensity value (∼50× the bulk concentration) was used for both neurotransmitters. *Black circles* depict the site III binding pockets.

Initial simulations of the R_6_ insulin hexamer with six dopamine molecules located in the phenolic pockets indicated that dopamine does not bind strongly to the phenolic pocket. At the same time, several dopamine molecules left the phenolic pocket to bind to the hitherto unexplored site III at the surface of the insulin hexamer. Therefore, an additional set of calculations was performed to characterize the binding of neurotransmitters to the surface of the insulin R_6_ hexamer (for simulation details, see the supplemental information). These simulations showed that dopamine binds at three equivalent binding sites in between the symmetry-related dimers (Fig. 5, *top*), which correspond to the previously sporadically observed site III. Analogous MD simulations for serotonin showed a similar mode of binding in site III but with a spatially looser neurotransmitter distribution, which reflects its weaker binding to this site compared with dopamine (Fig. 5, *bottom*). Finally, the binding of neurotransmitters at the surface sites has only a minor effect on the structure of the hexamer.

**Figure 6.**
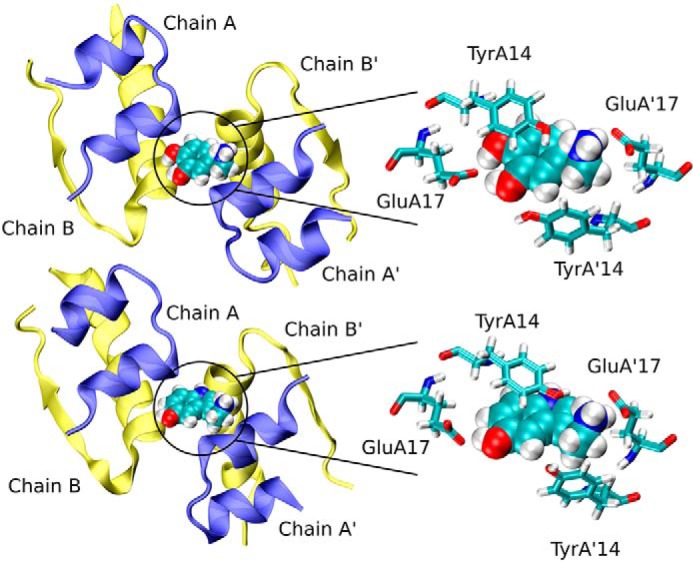
**Serotonin (*top*) and dopamine (*bottom*) binding site III formed by two adjacent insulin monomers (Chain A/A′ and Chain B/B′).** Detailed structures of amino acids involved in binding (Glu^A17^ and Tyr^A14^) are also shown.

**Figure 7.**
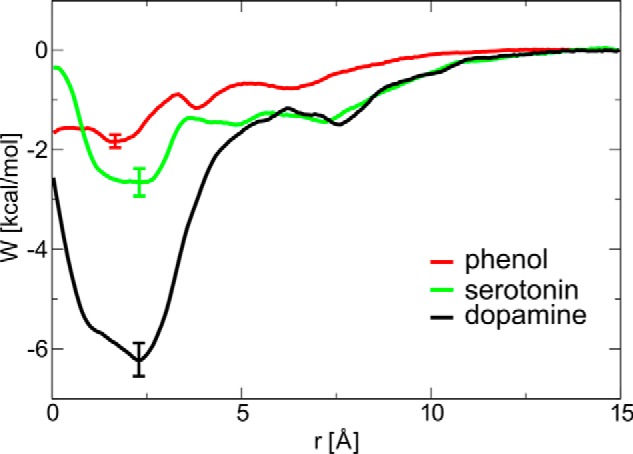
**Free energy profiles of phenolic ligands entering the surface binding site III.**

Both dopamine and serotonin bind in site III in a pocket between Glu^A17^ and Tyr^14^ from neighboring dimers (Fig. 6). The stronger binding of dopamine over serotonin shown by free energy calculations (see below) results from its interactions with Tyr^A14^, a hydrogen bond of the phenolic OH to Glu^A17^, and a salt bridge between the aminoethyl group and the carboxylic group of Glu^A17^. Similar interactions are present for serotonin; however, the steric fit is not as good as for dopamine. Dopamine, serotonin, and phenol binding in site III were quantified by evaluation of the binding free energies and the corresponding dissociation constants (Fig. 7 and Table 2) using the umbrella sampling method, with symmetry and volume entropy corrections. In contrast to site I, dopamine is the strongest binder to site III (*K_d_* 4.38 × 10^−3^ m), with serotonin and phenol showing a much weaker affinity for this surface site (*K_d_* of 2.13 × 10^−1^ m and 2.59 × 10^−1^ m, respectively).

**Table 2:** **Standard free energies of binding of phenol, dopamine, and serotonin molecules to the binding site III and the corresponding dissociation constants** *W*_0_ is free energy difference directly from umbrella sampling calculations, 〈‹Δ*G*_symm_〉 is hexamer symmetry contribution, Δ*G*_vol_ is volume entropy contribution (with respect to the standard state at 1 m), Δ*G_b_*° is overall standard free energy of binding, and *K_d_* is the corresponding dissociation constant.

Ligand in the binding site III	*W*_0_	〈‹Δ*G*_symm_〉	Δ*G*_vol_	Δ*G_b_*°	*K_d_*
	*kcal/mol*	*kcal/mol*	*kcal/mol*	*kcal/mol*	*m*
Phenol	−1.83 ± 0.28	−0.36	1.38 ± 0.22	−0.81 ± 0.36	2.59 × 10^−1^
Dopamine	−6.23 ± 0.67	−0.36	3.2 ± 0.2	−3.24 ± 0.70	4.38 × 10^−3^
Serotonin	−2.65 ± 0.55	−0.36	2.0 ± 0.1	−0.92 ± 0.56	2.13 × 10^−1^

### Insulin-serotonin crystal complex

**Figure 8.**
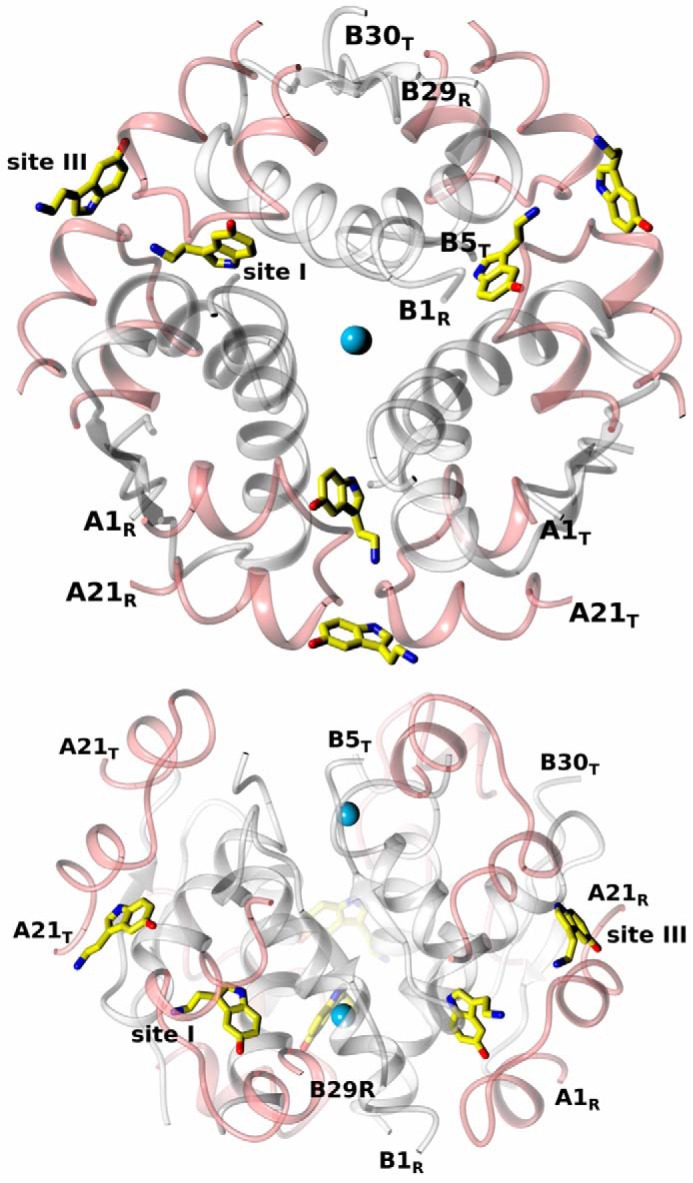
**Serotonin binding sites in insulin InsSerT_3_R_3_ hexamer: top view (*top*), side view (*bottom*) of the hexamer (A-chains in *pink*, B-chains in *white*, Zn^2+^ ions as *blue spheres*).** Sites I and III are indicated; some N and C termini of A/B-chains (T-state: T, R-state: R) are shown. Serotonin in atom-colored coded, with C-atoms in *yellow*.

The crystal structure of insulin grown in the presence of serotonin and Zn^2+^ revealed a T_3_R_3_ hexamer (referred to here as InsSerT_3_R_3_), with six neurotransmitters and two Zn^2+^ ions per hexamer (Fig. 8). The minimum serotonin concentration that still yielded complex crystals was within the 35–40 mm range. The asymmetric unit of this crystal contains 14 molecules of insulin, hence providing reliable, independent multicopy structural evidence. The hexamer quaternary structure is indeed T_3_R_3_ as the B1-B3 N termini acquire here a more R-like (fully α-helical) fold rather than an R^f^, “frayed,” conformation observed in T_3_R^f^_3_ oligomers.

Coordination of both Zn^2+^ ions is tetrahedral regardless of the R or T protein environment with axial Cl^−^ ligands. The overall fold of InsSerT_3_R_3_ is very similar to other T_3_R^f^_3_ hexamers. For example, the r.m.s.d. of InsSerT_3_R_3_ from complexes with phenol (PDB ID 1MPJ Ref. 29), Tylenol (PDB ID 1TYL; Ref. 23), paraben (3 MTH; Ref. 29), and 4-hydroxy-benzamide (PDB ID 1BEN; Ref. 25) are 0.8911 Å, 0.9886 Å, 0.9105 Å, and 1.1570 Å, respectively. Also, this hexamer is not significantly different from phenol-free T_3_R^f^_3_ hexamers induced by ions such as SCN^−^ (PDB ID 2TCI, r.m.s.d. 0.9919 Å; Ref. 29) and Cl^−^ (PDB ID 1G7A, r.m.s.d. 0.7765 Å (Ref. 38).

**Figure 9.**
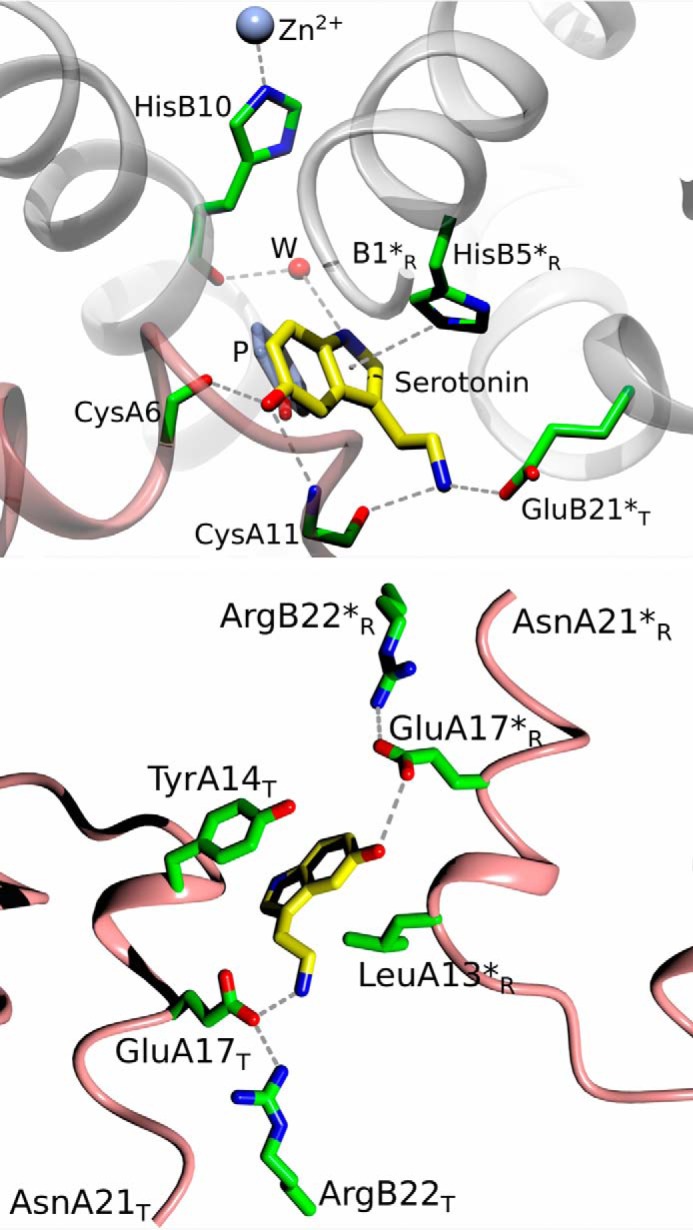
**Serotonin binding sites: *top*, site I; *bottom*, site III, in insulin InsSerT_3_R_3_ complex (insulin color-coded as in Fig. 7, with side-chain C-atoms in *green*, water as a *red sphere*).** *Dashed lines*, *HB*; *R and *T, correspond to symmetry-related dimer and T/R state of the monomer, respectively. Strong His^B5^-Nϵ2·π-pyrrole-center contact is also shown.

Serotonin occupies six sites in InsSerT_3_R_3_: three phenol “main” sites I and three hexamer surface sites III. It is tethered (Fig. 9) into site I via (typical for phenolic ligands) HBs of its OH group to -CO of Cys^A6^ (range of 2.41–2.43 Å) and to -NH of Cys^A11^ (2.93–3.17 Å); a weaker HB -OH contact to -CO of Ser^A9^ (3.38–3.41 Å) further stabilizes this phenolic anchor. A significant His^B5^-Nϵ2·π pyrrole HB (3.46–3.56 Å) contributes to the immobilization of the serotonin indole ring, which is assisted further by more subtle van der Waals (hydrophobic) interactions, especially with Leu^B17^ (3.45–3.59 Å). The aminoethyl side chain of serotonin points toward the surface of the hexamer, *i.e.* into the space/gap between two 3-fold related dimers. The -NζH amino group of this side chain is hydrogen-bonded to Oϵ1 of Glu^B21^ (2.55–2.75 Å) and -CO of Cys^A11^ (2.72–2.79 Å).

In contrast to site I, the serotonin-binding mode to site III is rather dynamic, indicating its mobility on the InsSerT_3_R_3_ surface. Although serotonin is pincered there in a hydrophobic clamp of Tyr^A14^ (π·π interactions ∼3.5 Å) and the side chain of Leu^A13^ (from a symmetry related dimer), the ligands' electron density is generally less well defined there. The serotonin phenolic OH group makes hydrogen bond to the Oϵ atoms of Glu^A17^ (2.96–3.44 Å), and it can be stabilized by water-mediated HB to the hydroxyl of Tyr^A14^. The HBs of the Glu^A17^ carboxylic side chain to the guanidinium group of Arg^B22^ (2.75–2.82 Å) contributes further to the structural stability of the serotonin-OH environment. However, it is evident that the aromatic moieties of Tyr^14^ and serotonin can slide parallel to each other, indicating further adaptability of this mode of binding. Relative flexibility of site III is also seen in different modes of the HB network to the -Nζ group of the serotonin aminoethyl side chain. It is engaged primarily by HBs to the carboxyl side chain of Glu^A17^ (2.65–2.76 Å) and via a water molecule to Arg^B22^, but this network of HBs is prone to disruption due to the flexibility of the serotonin surface-exposed side chain. In summary, the Glu^A14^/Arg^B22^-pair are the 2-fold symmetry-related providers of HBs to serotonin (one pair to the ligand's OH group, the other one to the end of its side chain) in site III, but these HB networks are not fully symmetrical in overall geometry and strength due to a non-symmetrical ligand in this site. The swing of the other (not involved in serotonin binding) Tyr^A14^ side chain, away from site III, breaks down the 2-fold symmetry of this interface even further. Finally, we note that all our dopamine/insulin co-crystallizations were unsuccessful, likely due to the oxidation of the ligand, despite the inclusion of some anti-oxidation agents in the media.

### Insulin-serotonin-arginine crystal complex

A similar set of crystallization conditions yielded crystals of insulin in a ternary complex with serotonin and arginine (referred to here as InsSerArgT_3_R_3_). Arginine- and Zn^2+^-containing crystallizations of insulin were carried out in the presence and absence of the previously established optimum serotonin concentration (40 mm). Serotonin-arginine-containing solutions yielded several morphologically different, but crystallographically very isomorphous, crystal forms. Despite their similarity, two of these forms (f1, f2) (referred to as InsSerArgf1-T_3_R_3_ and InsSerArgf2-T_3_R_3_) are reported here due to a dynamic (*i.e.* with high level of disorder) nature of Arg/insulin binding, hence the need for more independent structural evidence about the nature of these interactions.

Both InsSerArg complexes appear in the T_3_R_3_ hexamer state, with all Zn^2+^ ions in a tetrahedral coordination with Cl^−^ as an axial ion. They are very similar to the InsSerT_3_R_3_ complex, with overall r.m.s.d. values of 0.2193/0.2349 Å between these structures.

**Figure 10.**
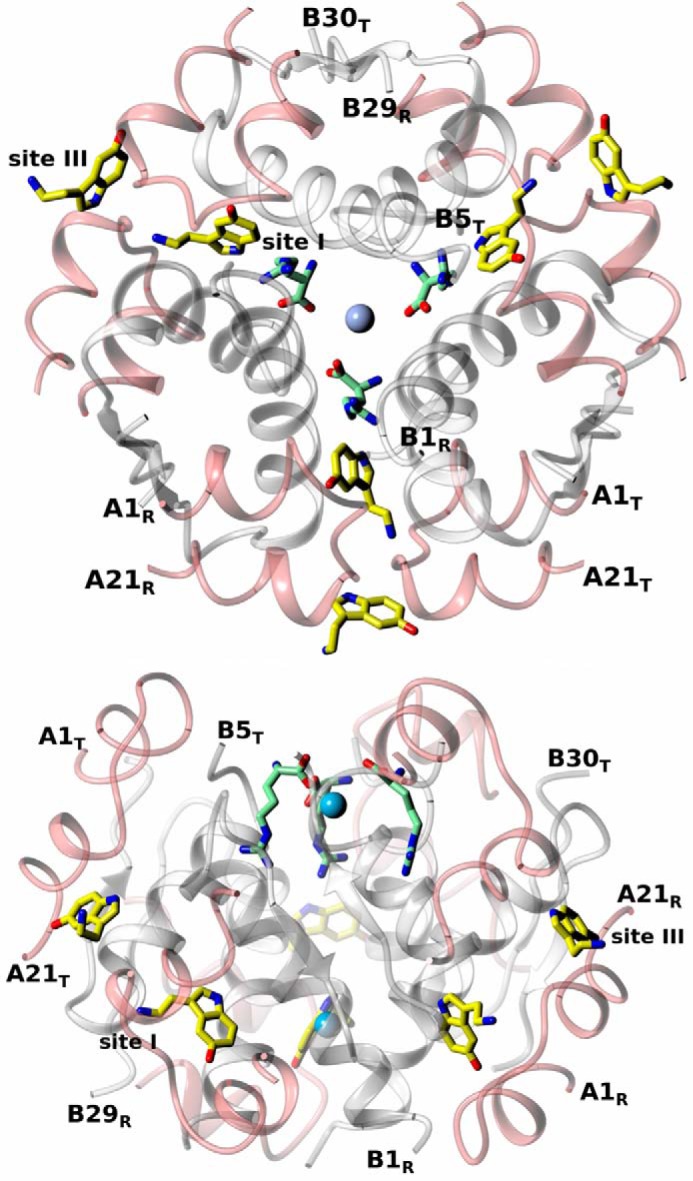
**Serotonin and arginine binding sites in insulin InsSerArgT_3_R_3_ hexamer: top view (*top*) and side view (*bottom*) of the hexamer (labeling and color-coding as in Fig. 7, with arginine C-atoms in *light green*).**

Despite the presence of arginine, the serotonin modes of binding in sites I are practically the same as in the Ins-Ser complex (Fig. 10). However, the site III shows more significant structural variety in binding of this neurotransmitter. Some serotonins in site III are bound in a fashion observed in InsSerT_3_R_3_; however, some of these ligands are flipped in both InsSerArgT_3_R_3_ crystal forms, *i.e.* the -OH and aminoethyl groups switch their positions. As both of them benefit from a similar HB network of symmetrical Glu^A14^/Arg^B22^ tandems, the overall HB-connectivity is conserved here as well, with the exception of the Nζ atom of the aminoethyl side chain. It forms in some sites III a new HB with the hydroxyl group of Tyr^A14^ (2.67 Å) besides maintaining a HB to the side chain of Glu^A14^ as well.

It has to be stressed that several serotonins in site III and arginine molecules (see below) in the InsSerArgT_3_R_3_ complex have been refined with half-occupancies and, in some cases, with more arbitrary modeling due to their partial definition in the electron density maps. This reflects the dynamic and mobile character of some ligand/hormone interactions in InsSerArgT_3_R_3_ hexamers.

**Figure 11.**
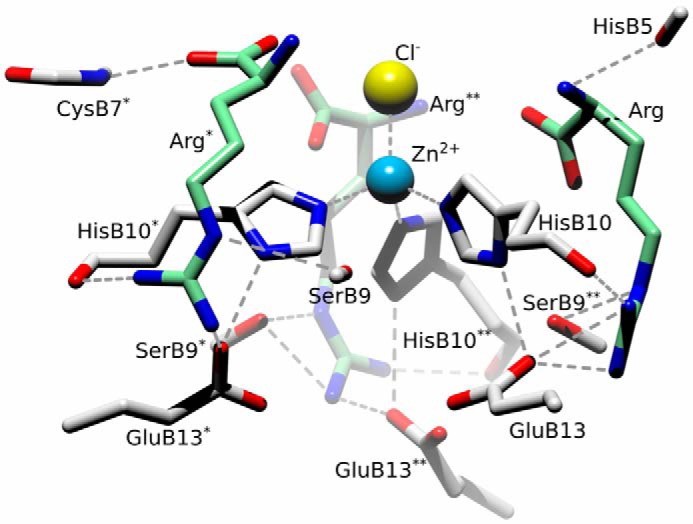
**Arginine binding sites in the insulin InsSerArgT_3_R_3_ hexamer.** Labeling and color-coding is as in Fig. 8, with Cl^−^ ion as *yellow*.

Arginine molecules occupy the “T-state-half” of the trimer (Fig. 11), with their Cα moieties being part of the T-state trimer surface (on the level with the Cl^−^ ion), whereas their side chains point toward the hexamer core, relatively parallel to its central axis. The aminocarboxy-Cα groups of Arg have been modeled in both InsSerArgT_3_R_3_ forms with some flipped/alternative conformations around the Cα atom.

The dominant stabilizations of the Arg Cα-end result from HBs of the carboxyl group to the NH of Cys^B7^ (2.76–3.31 Å) and amino acid amine group to CO of His^B5^ (2.86–3.18 Å). However, it is possible to model this part of arginine with alternative conformations, a likely effect of a dynamic character of these ligands at the wider, and more solvent-accessible, T_3_ side of the hexamer.

In contrast, the HBs of the Arg guanidinium groups are much better defined, especially in the InsSerArgf2T_3_R_3_ form. One of their main signatures is the extensive HB network with both Glu^B13^ and His^B10^ residues. Here, the Glu^B13^ Oϵ1/Oϵ2 atom forms HBs to Arg Nη1 (2.11–2.33 Å) and Nδ1 of His^B10^ (2.82–3.14 Å), whereas the second Nη2 atom of the guanidinium group is hydrogen-bonded to CO of His^B10^ as well (2.71–2.82 Å). The last guanidinium Nϵ1 atom locks this group by HB to the hydroxyl Oγ of Ser^B9^. Some of these HBs are broken in the InsSerArgf1T_3_R_3_ form, also indicating the increased mobility of this environment, especially some shifting of the arginine parallel to the hexamer 3-fold axis.

### Determination of ligand K*_d_* values by solution 4H3N assay

The interactions of insulin hexamers with phenol, serotonin, dopamine, and arginine in solution were investigated and quantified by spectroscopic studies, with the 4H3N chromophore, which binds exclusively to His^B10^ sites in the R-state only, which is associated with the red shift of its absorption spectrum. This allows for the determination of the apparent binding constants (*K_d_*) of selected ligands to insulin hexamers and estimation of the values of their maximum specific binding (B_max_)_,_ which can be considered here as a measure of the amount of the R state induced by the ligand. In addition, the Hill coefficients (*h*), which indicate the scale of cooperativity (likely very complex) between ligand and/or insulin its hexameric state upon ligand binding, were evaluated as well.

**Figure 12.**
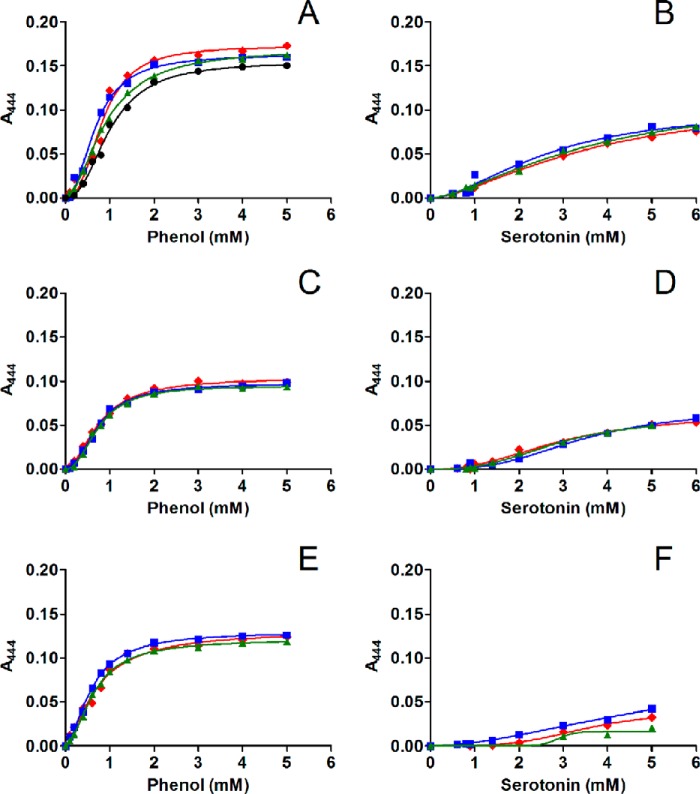
Titration of insulin hexamers and 4H3N with phenol (*A*) or serotonin (*B*). Titration of insulin hexamers and 4H3N preincubated with 5 mm arginine with phenol (*C*) or serotonin (*D*). Titration of insulin hexamers and 4H3N preincubated with 5 mm dopamine with phenol (*E*) or serotonin (*F*). All measured binding curves are shown.

First, we determined the change in 4H3N absorption upon titration of selected ligand to T_6_ insulin hexamer preincubated with this chromophore. Fitting of all curves (Fig. 12) gave *K_d_*, B_max,_ and *h* values (Table 3) for all types of experiments. The data from Table 3 are also presented in a bar plot in supplemental Fig. S2. The 4H3N spectroscopic data indicate that phenol is the strongest binder to insulin hexamers, about 4× stronger than serotonin (*K_d_* values of 0.85 mm and 3.34 mm, respectively; see Table 3). In contrast, titration of the same T_6_ insulin hexamers by arginine and dopamine did not induced any changes in the 4H3N absorption spectra (data not shown), indicating a lack of binding to site I and, subsequently, inability to induce T_6_→T_3_R_3_/R_6_ transitions.

**Table 3:** **Values of *K_d_*, *B*_max_, and Hill coefficient (*h*) for the interaction of phenol or serotonin with insulin hexamers in the presence of 4H3N and/or arginine and serotonin**

Ligand	*K_d_* ± S.D.	B_max_± S.D.	*h* ± S.D.
	*mm*		
Phenol (*n* = 4)	0.86 ± 0.13	0.17 ± 0.01	2.22 ± 0.30
Phenol and 5 mm Arg (*n* = 3)	0.75 ± 0.01	0.10 ± 0.00	2.07 ± 0.19
Phenol and 5 mm dopamine (*n* = 3)	0.64 ± 0.06	0.13 ± 0.01	1.68 ± 0.18
Serotonin (*n* = 3)	3.34 ± 0.52	0.11 ± 0.08	1.63 ± 0.10
Serotonin and 5 mm Arg (*n* = 3)	3.12 ± 0.31	0.06 ± 0.00	2.64 ± 0.27
Serotonin and 5 mm dopamine (*n* = 3)	3.93 ± 1.28	0.05 ± 0.04	7.12 ± 7.50

The addition of phenol or serotonin to insulin/4H3N that was also preincubated with 5 mm Arg resulted, however, in different patterns of binding curves (Fig. 12, *C* and *D*), compared with sole ligands-insulin/4H3N titrations (Fig. 12, *A* and *B*). Similar trends were observed for phenol and serotonin titrations of insulin/4H3N preincubated with 5 mm dopamine (Fig. 12 *E* and 11*F*). It seems that in both cases the preincubation of insulin/4H3N with arginine or dopamine (which are not hormone Site I binders on their own) lowers the phenol *K_d_* to 0.75 and 0.64 mm, respectively. In contrast, arginine and dopamine do not change the binding affinity of serotonin to insulin hexamers (Table 3, Fig. 12*A*).

The higher B_max_ value for phenol than for serotonin (0.17 *versus* 0.11) may also indicate that serotonin is not able to fully induce the R_6_ state of the hexamers. Note that preincubation of insulin/4H3N with arginine or dopamine lowers the B_max_ values of phenol or serotonin binding to insulin (Fig. 12*B*). The Hill coefficient of phenol binding to insulin hexamers (Fig. 12*C*) is also higher (*h* = 2.2) than that for serotonin (1.6), indicating a lower degree of cooperativity for serotonin-insulin interactions. Interestingly, the presence of arginine or dopamine lowers cooperativity (*i.e.* the *h* value) of phenol-insulin binding but increases the *h* value for serotonin interactions with the hormone. This effect seems to be even more noticeable in the case of dopamine (*h* = 7.1), but these data are accompanied by a high experimental error (see Table 3 and supplemental Fig. S2).

## Discussion

Our parallel and independent MD simulations, X-ray crystallography, and ligand binding experiments provide a concise picture showing that the insulin hexamer can accommodate and be affected by ligands that are present in the hormone storage LDCV granules in pancreatic β-cells. These ligands can be of endogenous origin, such as neurotransmitters (serotonin or dopamine) involved in regulation of insulin secretion, or they can be byproducts of pro-hormone/pro-insulin processing (*e.g.* arginine) that accumulate in the granules.

All the above methods indicated that serotonin is a good phenol-compatible ligand for insulin hexamer site I, whereas dopamine is not a site I binder. Serotonin-site I binding engages its phenolic OH in “phenol-canonic” HBs to CO of Cys^A6^ and NH of Cys^A11^. This is despite an ∼29° tilt of its indole plane with respect to the phenol benzene ring, a movement of the aromatic moiety that is also observed (to a different degree) in Tylenol (23), paraben (29), and benzamide (25) complexes. However, the side chains of the latter phenolic ligands are oriented toward the central channel of the hexamer, whereas the aminoethyl group of serotonin points into the opposite direction, *i.e.* toward the surface of the hexamer. This results in further enhancement of serotonin stability in site I by fixing its side chain via HBs with the CO of Cys^A11^ and carboxyl of Glu^B21^. The fitting of serotonin in site I is completed with a strong His^B5^-Nϵ2·π-pyrrole contact. The firmness of serotonin-site I interactions, which is propagated likely toward the N termini of the B-helix, combined also with a very good overall fit of serotonin into site I may be behind the induction of the R (*i.e.* not R^f^) conformation of the T_3_R_3_ hexamer. This is rather unique, as this type of insulin quaternary arrangement occurs mainly in the T_3_R^f^_3_ state. T_3_R_3_ hexamers were observed only within a R_6_→T_3_R_3_ retrograde conformational change (39–41) upon evaporation of phenol from R_6_ insulin crystals, which is a process that cannot occur for the non-volatile serotonin ligand. Therefore, the stability of the SerInsT_3_R_3_ complex may indicate that this hormone's conformer is indeed close to its putative native form in pancreatic LDCV (as postulated, for example, by Dunn (26)). Moreover, larger B_max_ and Hill coefficient *h* values (obtained from 4H3N spectroscopy studies) for phenol as compared with serotonin suggest that the latter, despite its “perfect” fit into site I, cannot fully induce the insulin R_6_ state (as also confirmed by the lack of R_6_ serotonin hexamer crystals even at a very high ligand concentration). These findings further corroborate the potential physiological aspect of the T_3_R_3_ insulin conformer as the only possible oligomeric species in the presence of serotonin. Although MD simulations did not exclude the presence of a stable SerInsR_6_ conformer, the R_6_ state was postulated in the MD work *a priori* (due to its thermodynamic superiority in the presence of phenol ligands) to identify the potential ligand-binding sites rather than to investigate the impact of a particular ligand on the state of the insulin hexamer.

The lack of stable site I-dopamine complexes was demonstrated by all methods employed in this study. It seems that this is not due to a steric ligand-cavity incompatibility, as the dopamine dispersion contribution to free energy of binding in the MD simulations is more negative compared with phenol. Rather, as the superposition of dopamine with phenol or serotonin ligands in site I suggests, the lack of dopamine binding is due to an electrostatic mismatch, namely its inability to accommodate the charged aminoethyl side chain in the site I cavity. The smaller aromatic scaffold of dopamine thus does not allow its side chain to reach the hexamer surface and form HBs observed in serotonin complexes.

As the 4H3N spectroscopic applicability was limited here to the environment of His^B10^ and, hence, the site I, the evidence for other insulin site-ligand interactions came solely from MD simulations and X-ray crystallography. Both showed serotonin binding to surface site III, whereas only MD calculations predicted the possibility of a stable site III-dopamine complex (note, however, that dopamine-insulin co-crystallization may be hampered by a rapid chemical degradation/oxidation of dopamine). Interestingly, MD calculations indicated superior dopamine binding to site III, with its *K_d_* ∼50 times better than for phenol or serotonin. MD-predicted dopamine dominance at site III is interesting, as these simulations show its binding in a similar mode to serotonin-site III complex, which is observed both in MD simulations and crystal structures. Both neurotransmitters engage Tyr^A14^ and Glu^A17^/Arg^B22^ side chains from 2-fold symmetry-related dimers in the network of HB and π·π-stacking interactions. Moreover, crystal structures of InsSerT_3_R_3_ also underline the dynamical character of site III mode of binding, which involves flipping of the serotonin indole ring (*i.e.* swapping positions of -OH and the aminoethyl side chain) and a certain variability of the Tyr^A14^-OH/Glu^A17^/Arg^B22^ HB patterns.

The impact of these ligands, which are natural components of pancreatic β-cells insulin-storing LDCVs, was also extended from neurotransmitters to probing of arginine-insulin hexamer interactions. It was postulated earlier that arginine can accumulate in these granules upon processing of pro-insulin at its two sites (Arg-31–Arg-32 and Lys-64–Arg-65) (34, 35), which are cleaved by specific convertases during maturation of this hormone (42, 43). Here, the X-ray crystallography revealed that arginine can penetrate serotonin-containing hexamers (InsSerArgT_3_R_3_) without significant changes to their InsSerT_3_R_3_ state. Arginine is accommodated there in channels within the T_3_ trimer, parallel to the hexamer 3-fold axis and opposite to R_3_ trimer sites I filled by serotonin, which leads to filling the voids around Zn^2+^ and His-B10. Binding of arginine in InsSerArgT_3_R_3_ is dynamic with high mobility and alternative, slightly different ligand conformations. The flexibility of arginine is concentrated mostly around its -CαNH_2_COO^−^ moieties, which form weak alternative HB to main chain groups of Cys^B7^ and His^B5^, also exhibiting some flipping around the Cα atom. In contrast, the guanidinium groups of the arginines are firmly engaged in extensive networks of HBs, which link them with side chains of Glu^B13^, the side- and main-chain of His^B10^, and the side chain of Ser^B9^. This simultaneous binding of the two very different ligands to the T_3_R_3_ insulin hexamer polarizes this oligomer even further into two structurally distinct trimers, Arg_3_T_3_ and Ser_6_R_3_. Although Arg binding in the ArgT_3_ trimer has a notable impact on the stability of the usually mobile Glu^B13^ side chains, the solvent-exposed flexible -CαNH_2_COO^−^ moieties may contribute to the disorder of the N-terminal parts of B-helices, which is, however, untraceable up to the His^B5^ site in both arginine-containing and arginine-free serotonin complexes. In contrast, the Glu^B13^ side chains are fully disordered in the Ser_6_R_3_ trimer, whereas the serotonins in sites I facilitate fixing of the B1-B19 helices in the R-state.

The structural partition of Arg_3_T_3_ and Ser_6_R_3_ is more complex. First, although the site I pocket is mainly formed by two R-monomers, the T-state monomer also contributes here by Leu^B17^ and Glu^B21^ toward serotonin aromatic rings and side-chain binding, respectively. Secondly, serotonin surface site III also mediates the structural interface between T_3_ and R_3_, as this binding site (formed by Tyr^A14^/Glu^A17^) is provided by the T- and R-monomers from the 2-fold symmetry related dimers.

Interestingly, 4H3N spectroscopic data indicate that the presence of arginine increases (∼13%) phenol (but not serotonin) affinity for site I and also increases the cooperative character of serotonin binding (as demonstrated by a higher Hill *h* factor). These structural and spectroscopic data provide evidence for the T_3_-R_3_ trimers cross-talk and, as postulated previously, for a heterotropic allostery within the insulin hexamer (22, 30, 44–47). However, the exact molecular detail behind this phenomenon has been elusive, likely involving a network of propagating longer range interactions, which fit the Seydoux, Malhotra, and Bernhard (SMB) cooperativity model (48). The stabilizing effect of guanidinium groups on Glu^B13^-His^B10^ and Zn^2+^ may be one of the initial steps in this process, priming the other half of the hexamer for more effective serotonin binding (*i.e.* an element of the so-called half-site reactivity). The stability of InsSerArgT_3_R_3_ and InsSerT_3_R_3_ hexamers in the presence of physiological ligands, *i.e.* trapping of these oligomers in a stable T_3_R_3_ state, agrees also with the finding that conformational fluctuations of the T_3_ trimer needed for the appearance of site I become constrained upon formation of T_3_R_3_ (49). This can be considered as one of the features of the negative cooperativity effect in the insulin hexamer, predicted by the SMB model as well.

The present 4H3N spectroscopic data also suggest a dopamine-lowering effect of phenol's *K_d_*. As there is no evidence for dopamine site I binding, this allosteric effect may result from longer-range cross-talk of the dopamine-site III complex with the environment of site I. On the other hand, dopamine does not seem to significantly change the serotonin *K_d_*, and therefore, some “competitive” crowding effect (*e.g.* dynamic occlusion of site I) between these two neurotransmitters cannot be excluded. It may also mean that dopamine-site III binding facilitates the diffusion of a smaller ligand (*i.e.* phenol) into site I, whereas the increased stability of the insulin R_3_-like trimer (via new HBs and interactions in site III) may obtrude its effect on binding of a larger ligand (such as serotonin).

It is worth mentioning that several tubular electron densities are also observed in InsSerArgT_3_R_3_ on the top of the T_3_ trimer. These are linked with the modeled arginine ligands, suggesting the presence of more of these molecules on the hexamer surface, in the form of a hydrogen-binding network of ligands. Some of this electron density is in contact with the T_3_ Zn^2+^ ion. Therefore, a contribution of the arginine -COO^−^ group to the tetrahedral/monodentate coordination of Zn^2+^ (observed also in the insulin R_6_ structure; Ref. 41) cannot be excluded either. This direct arginine-Zn^2+^ interaction would hamper the T_3_→R_3_ transition even further, thus reinforcing the stability of the T_3_R_3_ hexamer.

Arginine is widely used *in vitro* as a nonspecific protein-folding stabilizing agent (49, 50), and so its presence in InsSerArgT_3_R_3_ structures may reflect some of its physiologically relevant roles as a ubiquitous insulin ligand. First, it may serve as an unspecific, granules-abundant, insulin-folding stabilizer and hexamer half-life enhancer. Second, it may act as a more specific endogenous ligand that modulates the hexamer cooperativity, also enhancing the stability of its particular T_3_R_3_ storage form. This more specific role of arginine may result, among others, from its guanidinium counterion-like neutralization of the repulsive system of the Glu^B13^ side chains in the hexamer core. Moreover, a simultaneous arginine-mediated cross-linking of Glu^B13^ side chains with Zn^2+^-binding His^B10^, leading to stabilization of this structurally key region, further exemplifies the ligand-like role of this amino acid.

It has to be stressed that insulin crystallization in the granules can be also affected by the intragranular Zn^2+^ concentrations, which are modulated by some Zn^2+^ transporters (ZnT), especially by the disease-associated ZnT8 variants (51). However, the focus here was on insulin/neurotransmitter interactions, hence the experimental Zn^2+^ concentrations (0.4–8 mm) were maintained within typical insulin *in vitro* crystallization ranges. Interestingly, insulin crystallized here only in the form of 2Zn^2+^ hexamers, regardless the very high (25:1) Zn^2+^-hormone molar excess in the crystal-yielding conditions.

There are ongoing efforts for a better insight into the size and content of insulin storage granules (*e.g.* Ref. 52). Their exocytosis can also be morphologically and mechanistically quite heterogeneous (so-called “kiss-and-run” phenomenon), releasing varying amounts and forms of granule content and on different time scales (53). Therefore, the final amount and form of insulin discharged to the circulation can be a very complex and multifactorial process; whether insulin-neurotransmitter-arginine hexamers are one of its variables remains to be seen.

## Conclusions

We have demonstrated here that insulin oligomeric forms in storage granules in β-pancreatic cells could be regulated by certain endogenous components of these vesicles, such as serotonin, dopamine, and arginine. They are able to (i) shift the insulin oligomeric equilibrium toward the T_3_R_3_ state, which therefore may be considered as the insulin storage form in pancreatic β-cells, (ii) affect and modulate the allostery of the insulin hexamer, (iii) may provide folding stability and protection for the insulin hexamer, and consequently (iv), may have a direct role in the modulation of insulin release from β-cells *in vivo*. Moreover, these ligands can act in a synergistic, heterotropic fashion, pointing to a co-operative, complex, and dynamic nature of the interactions within the insulin hexamer.

Therefore, the importance of the neurotransmitters for insulin beta-cell biology can be either cumulative or even independent. Serotonin/dopamine are involved in the regulation of exocytosis of the granules, whereas their impact on insulin storage can be seen as a serendipitous side effect of the evolution of the hormone within the β-cell environment and its optimization for a particular, species-specific, physiological profile.

It is also tempting to consider serotonin/dopamine-human insulin interactions as molecular fingerprints of insulin evolution in the animal kingdom, as insulin-like hormones are expressed in neurons in many invertebrates (54). The newly discovered (and still puzzling) role of insulin in the central nervous system and the emergence of neurodegeneration-linked so-called type III diabetes thus places our findings in a much wider physiological context (55, 56).

In summary, we show here that the insulin hexamer can act as a macromolecular sponge, keen to bind and assimilate a variety of physiological ligands and ions. This suggests that clinical exploration and use of insulin-hexamer-stabilizing ligands, driven by steering of the pharmacokinetics of this hormone, may mirror its physiological canonical properties, rooted also in insulin evolution. Although the present work is aimed at shedding new light on the physiological storage state of this hormone, it may also open ways to novel approaches in medical formulations of insulin, toward its more desired closer to *in vivo* profile during subcutaneous injections administered in diabetes.

## Experimental procedures

### Molecular dynamics simulations

All MD simulations were performed using the AMBER 14 program (57, 58). In all simulations the AMBER ff03 protein force field (59) and SPC/E water model were used (60). Production simulations were performed in the isothermal-isobaric ensemble at ambient conditions of *T* = 300 K and *p* = 1 atm using the Berendsen barostat and thermostat (61). The only exception was thermodynamic integration calculations, where the temperature was controlled by a Langevin thermostat with a reference temperature of *T* = 300 K and a collision frequency of 5 ps^−1^ in order to avoid problems of non-ergodicity when the ligand is fully decoupled from its environment. 3D periodic boundary conditions were applied with a non-bonded interaction cutoff of 9 Å. The long-range electrostatic interactions were accounted for using the particle mesh Ewald method (62) using a cubic spline interpolation. The density of the charge grid was 64 × 64 × 64, and the direct sum threshold was 10^−5^. Van der Waals interactions beyond the cutoff were treated using the continuum model correction for energy and pressure. All bonds containing hydrogen atoms were constrained using the SHAKE algorithm (63). A time step of 2 fs was employed.

Mildly acidic conditions (pH ∼ 5.5) of insulin storage granules would generally tend to favor a fully protonated side chain of His^B5^ (64). However, due to zinc coordination the Nϵ2 atom of His^B10^ is actually deprotonated (65, 66). In addition, the six Glu^B13^ side chains in the middle region of the insulin hexamer were considered as deprotonated. The overall charge of the protein is, therefore, −6. Initial simulations indicated diffusion of Na^+^ cation/cations into the middle region. At least one Na^+^ cation was always present in the middle region of the hexamer being placed to the middle region from the start of the simulation.

Substitution of the site I-bound phenol by the neurotransmitters serotonin or dopamine was also investigated. The R_6_-state insulin complex with six phenols in sites I (referred to here as InsPheR_6_) was used as a reference structure (PDB ID 1AIY) due to a higher stability of this conformer and homogenous saturation of insulin hexamer with the same ligand (67). The starting structures for simulations were obtained by exchanging phenol molecules in site I by either dopamine or serotonin molecules with two initial conformations for each ligand. The ligands were built from the phenol core (1 oxygen atom and 6 carbon atoms), the structure of which was available from X-ray structure). Subsequent restrained minimization eliminated any potential steric clashes. Starting geometries of phenolic ligands inside the phenolic pockets are depicted in Fig. 2.

Phenol, serotonin, and dopamine were assigned parameters from ff03 AMBER force field using the ANTECHAMBER package (68). Both serotonin and dopamine have a charge of +1 *e* at pH 5.5. Partial atomic charges were obtained by the RESP (Restrained Electrostatic Potential Fit) (83) method, calculated at the HF/6–31G+ level, and they are listed in supplemental Table S2, with the explanation in the supplemental Figure S5. These calculations were performed using the Gaussian 09 package (69).

The B10-Zn interaction potential had to be re-parametrized in order to account (at least partially) for the electronic polarization and charge transfer effects and thus to reproduce experimental data. The charges on zinc ions and on His^B10^ were modified according to the results from *ab initio* calculations on small model systems to +1.5 *e* and +0.1677 *e*, respectively. These were obtained using the RESP/NPA (Natural Population Analysis) (84) analysis employing the B3LYP/aug-cc-pvtz level of theory (85, 86). In a similar spirit, to account for electronic polarization effects in a mean-field way (70–73), we rescaled bulk Na^+^ and Cl^−^ (including the Na^+^ cation located in the middle of the hexamer) ionic charges by a factor of 0.75.

Each of the liganded insulin R_6_ hexamers was immersed into a unit cell containing 9000 SPC/E water molecules, with Na^+^/Cl^−^ ions added to acquire overall electroneutrality with no excess of salt present. After preparation, the energy of each of the systems was minimized using 5000 steps of the steepest descent method, where the protein and phenolic ligands were restrained with a harmonic potential. The systems were then subjected to 200 ps of isothermal-isochoric molecular dynamics, where the temperature was slowly raised from 10 to 300 K. This was followed by 1.2 ns of an isothermal-isobaric equilibration, which led to an equilibrated cell size of ∼69 × 68 × 66 Å^3^. Systems were assessed as equilibrated by monitoring temperature, cell size, density, and root mean square displacement of the protein. After equilibration, the production runs were propagated for 600 ns.

In addition to direct MD simulations free energy calculations employing the thermodynamic integration method were performed to determine apparent binding constants *K_d_* of phenol and the two neurotransmitters to the R_6_ hexamer. The free energy cycles and further details of these simulations are presented in the supplemental Figs. S6 and S7, and Table S3. Similar free energy calculations for evaluation of the *K_d_* values to ligand binding sites III were performed using the umbrella sampling methods (for more details see the supplemental information, and Table S4). Here, to stabilize the hexamer R_6_ state, site I was filled with phenol before the search for dopamine site III binding mode, as this neurotransmitter did not bind to site I in MD simulations.

### X-ray crystallography

Crystallizations of all insulin complexes reported here were performed with the in-house insulin crystallization screens that cover most of the previously reported crystal growth parameters. Crystallization conditions, data collection, refinement, and models statistics as well as PDB codes are provided in supplemental Table S1. All crystals were directly flash-cooled in liquid N_2_. X-ray data were collected at 100 K and processed by *xia2* (74), and model building and refinement (F>0σF) were performed by COOT (75) and the CCP4 suite of programs (76). Crystal structures were solved by Molrep (77) with the B1-B6-truncated insulin hexamer, hexamer-derived dimer, and insulin monomer as a model (based on PDB ID 1MSO; Ref. 78) and refined by Refmac 5.8 (79). Examples of electron density maps are shown in supplemental Figs. S3 and S4. The figures were made using CCP4mg (80). For structural comparisons, all insulin hexamer structures were superimposed in COOT by the SSM fit option. For comparison of the site I ligand-binding modes, the relevant dimers were superimposed by Cα matching of the B9–19 helix for one of the monomers by the LSQ option in COOT.

### Determination of ligand K*_d_* values by a solution 4H3N assay

Solution studies of the interactions of phenol, serotonin, dopamine, and arginine with porcine insulin were performed following the protocols by Huang *et al.* (81), Bloom *et al.* (82), and Huus *et al.* (46). An anionic ligand 4H3N was used as a sensitive chromophoric probe to determine ligand binding curves by monitoring UV-visible absorption. 4H3N binds only in the insulin R-state (in the vicinity of His^B10^), which red-shifts its absorption spectrum. The samples were dissolved in 10 mm potassium phosphate buffer at pH 7.4. In all measurements porcine hexameric Zn^2+^-insulin (two atoms of zinc per one hexamer) and 4H3N were used at 0.6 mm and 0.225 mm concentrations, respectively. Insulin was preincubated with 4H3N in a total volume 0.6 ml for 20 min at room temperature, after which a particular ligand (phenol, serotonin, dopamine, or arginine) was added in a minimum volume (0.6–3 μl) of 10 mm potassium phosphate buffer. After 15 min of reverse transcription equilibration, the UV-visible absorbance spectra were collected on a Perkin, Lambda 25 UV-visible Spectrometer. The spectra were taken in the range of 300–550 nm, and the difference spectra at λ_max_ of 444 nm were obtained by subtracting the absorbance of ligand-free 4H3N from the absorbance of ligand-bound 4H3N. The binding curves were analyzed using a method of non-linear regression and a fitting program considering one-site specific binding with a Hill slope using GraphPad Prism 5.0. The final *K_d_*, B_max_, and *h* values with standard errors were calculated from at least three independently determined binding curves for each system.

## Author contributions

V. P. carried out the MD simulations. C. M. V., M. K., and T. R. G, carried out the crystallographic experiments. K. K. and P. H. carried out the solution 4H3N assays. J. P. T. collected the crystallographic data. A. M. B., P. J., J. J., and L. Z. conceived the study, designed the experiments, and analyzed the data. A. M. B., P. J., and J. J. wrote the paper. All authors discussed the results and commented on the manuscript.

## Supplementary Material

Supplemental Data

## Footnotes

5 The abbreviations used are:

IR: insulin receptor
LDCV: large dense core vesicle
4H3N: 4-hydroxy-3-nitrobenzoic acid
InsPheR_6_: insulin R_6_-state hexamer
r.m.s.d.: root mean square deviation
HB: hydrogen-bonded.

## References

[1] Taniguchi C. M., Emanuelli B., and Kahn C. R. (2006) Critical nodes in signalling pathways: insights into insulin action. Nat. Rev. Mol. Cell Biol. 7, 85–96 16493415 10.1038/nrm1837

[2] Cohen P. (2006) Timeline: the twentieth century struggle to decipher insulin signalling. Nat. Rev. Mol. Cell Biol. 7, 867–873 17057754 10.1038/nrm2043

[3] Atkinson M. A., Eisenbarth G. S., and Michels A. W. (2014) Type 1 diabetes. Lancet 383, 69–82 23890997 10.1016/S0140-6736(13)60591-7 PMC4380133

[4] Taylor S. I., Accili D., and Imai Y. (1994) Insulin resistance or insulin deficiency: which is the primary cause of Niddm. Diabetes 43, 735–740 10.2337/diab.43.6.735 8194657

[5] Turner R. C., Hattersley A. T., Shaw J. T., and Levy J. C. (1995) Type-II Diabetes: clinical aspects of molecular biological studies. Diabetes 44, 1–10 7813802 10.2337/diab.44.1.1

[6] Giovannucci E., Harlan D. M., Archer M. C., Bergenstal R. M., Gapstur S. M., Habel L. A., Pollak M., Regensteiner J. G., and Yee D. (2010) Diabetes and cancer: a consensus report. CA Cancer J. Clin. 60, 207–221 20554718 10.3322/caac.20078

[7] Vigneri P., Frasca F., Sciacca L., Pandini G., and Vigneri R. (2009) Diabetes and cancer. Endocr. Relat. Cancer 16, 1103–1123 19620249 10.1677/ERC-09-0087

[8] Cohen D. H., and LeRoith D. (2012) Obesity, type 2 diabetes, and cancer: the insulin and IGF connection. Endocr. Relat. Cancer 19, F27–F45 22593429 10.1530/ERC-11-0374

[9] Arrieta-Cruz I., and Gutiérrez-Juárez R. (2016) The role of insulin resistance and glucose metabolism dysregulation in the development of Alzheimer's disease. Rev. Invest. Clin. 68, 53–58 27103040

[10] McKern N. M., Lawrence M. C., Streltsov V. A., Lou M. Z., Adams T. E., Lovrecz G. O., Elleman T. C., Richards K. M., Bentley J. D., Pilling P. A., Hoyne P. A., Cartledge K. A., Pham T. M., Lewis J. L., Sankovich S. E., et al (2006) Structure of the insulin receptor ectodomain reveals a folded-over conformation. Nature 443, 218–221 16957736 10.1038/nature05106

[11] Lemmon M. A., and Schlessinger J. (2010) Cell signaling by receptor tyrosine kinases. Cell 141, 1117–1134 20602996 10.1016/j.cell.2010.06.011 PMC2914105

[12] Adams M. J., Blundell T. L., Dodson E. J., Dodson G. G., Vijayan M., Baker E. N., Harding M. M., Hodgkin D. C., Rimmer B., and Sheat S. (1969) Structure of rhombohedral 2 zinc insulin crystals. Nature 224, 491–495

[13] Dodson G., and Steiner D. (1998) The role of assembly in insulin's biosynthesis. Curr. Opin. Struct. Biol. 8, 189–194 9631292 10.1016/s0959-440x(98)80037-7

[14] Mayer J. P., Zhang F., and DiMarchi R. D. (2007) Insulin structure and function. Biopolymers 88, 687–713 17410596 10.1002/bip.20734

[15] Ward C. W., and Lawrence M. C. (2011) Landmarks in insulin research. Front. Endocrinol. 2, 76 10.3389/fendo.2011.00076 PMC3356151 22654826

[16] Weiss M. A. (2009) The structure and function of insulin: decoding the TR transition. In Insulin and IGFs (Litwack G., ed.), pp. 33–49, Elsevier Academic Press, Inc., San Diego CA 10.1016/S0083-6729(08)00602-X PMC3297421 19251033

[17] Menting J. G., Whittaker J., Margetts M. B., Whittaker L. J., Kong G. K., Smith B. J., Watson C. J., Záková L., Kletvíková E., Jiráček J., Chan S. J., Steiner D. F., Dodson G. G., Brzozowski A. M., Weiss M. A., Ward C. W., and Lawrence M. C. (2013) How insulin engages its primary binding site on the insulin receptor. Nature 493, 241–245 23302862 10.1038/nature11781 PMC3793637

[18] Menting J. G., Yang Y., Chan S. J., Phillips N. B., Smith B. J., Whittaker J., Wickramasinghe N. P., Whittaker L. J., Pandyarajan V., Wan Z. L., Yadav S. P., Carroll J. M., Strokes N., Roberts C. T. Jr., Ismail-Beigi F., et al (2014) Protective hinge in insulin opens to enable its receptor engagement. Proc. Natl. Acad. Sci. U.S.A. 111, E3395–E3404 25092300 10.1073/pnas.1412897111 PMC4143003

[19] Derewenda U., Derewenda Z., Dodson E. J., Dodson G. G., Reynolds C. D., Smith G. D., Sparks C., and Swenson D. (1989) Phenol stabilizes more helix in a new symmetrical zinc insulin hexamer. Nature 338, 594–596 2648161 10.1038/338594a0

[20] Bentley G., Dodson E., Dodson G., Hodgkin D., and Mercola D. (1976) Structure of insulin in 4-zinc insulin. Nature 261, 166–168 1272390 10.1038/261166a0

[21] Smith G. D., Swenson D. C., Dodson E. J., Dodson G. G., and Reynolds C. D. (1984) Structural stability in the 4-zinc human insulin hexamer. Proc. Natl. Acad. Sci. U.S.A. 81, 7093–7097 6390430 10.1073/pnas.81.22.7093 PMC392083

[22] Brzović P. S., Choi W. E., Borchardt D., Kaarsholm N. C., and Dunn M. F. (1994) Structural asymmetry and half-site reactivity in the T to R allosteric transition of the insulin hexamer. Biochemistry 33, 13057–13069 7947711 10.1021/bi00248a015

[23] Smith G. D., and Ciszak E. (1994) The structure of a complex of hexameric insulin and 4′-hydroxyacetanilide. Proc. Natl. Acad. Sci. U.S.A. 91, 8851–8855 8090735 10.1073/pnas.91.19.8851 PMC44704

[24] Whittingham J. L., Chaudhuri S., Dodson E. J., Moody P. C., and Dodson G. G. (1995) X-ray crystallographic studies on hexameric insulins in the presence of helix-stabilizing agents, thiocyanate, methylparaben, and phenol. Biochemistry 34, 15553–15563 7492558 10.1021/bi00047a022

[25] Smith G. D., Ciszak E., and Pangborn W. (1996) A novel complex of a phenolic derivative with insulin: structural features related to the T→R transition. Protein Sci. 5, 1502–1511 8844841 10.1002/pro.5560050806 PMC2143491

[26] Dunn M. F. (2005) Zinc-ligand interactions modulate assembly and stability of the insulin hexamer: a review. Biometals 18, 295–303 16158220 10.1007/s10534-005-3685-y

[27] Ciszak E., and Smith G. D. (1994) Crystallographic evidence for dual coordination around zinc in the T3R3 human insulin hexamer. Biochemistry 33, 1512–1517 8312271 10.1021/bi00172a030

[28] Smith G. D. (1998) The phenolic binding site in T3R3f insulin. J. Mol. Struct. 470, 71–80

[29] Whittingham J. L., Edwards D. J., Antson A. A., Clarkson J. M., and Dodson G. G. (1998) Interactions of phenol and m-cresol in the insulin hexamer, and their effect on the association properties of B28 Pro → Asp insulin analogues. Biochemistry 37, 11516–11523 9708987 10.1021/bi980807s

[30] Rahuel-Clermont S., French C. A., Kaarsholm N. C., Dunn M. F., and Chou C. I. (1997) Mechanisms of stabilization of the insulin hexamer through allosteric ligand interactions. Biochemistry 36, 5837–5845 9153424 10.1021/bi963038q

[31] Falck B., and Hellman B. (1963) Evidence for presence of biogenic amines in pancreatic islets. Experientia 19, 139–140

[32] Lundquist I., Ekholm R., and Ericson L. E. (1971) Monoamines in pancreatic-islets of mouse 5-hydroxytryptamine as an intracellular modifier of insulin secretion and hypoglycemic action of monoamine-oxidase inhibitors. Diabetologia 7, 414–422 5004176 10.1007/BF01212056

[33] Ustione A., Piston D. W., and Harris P. E. (2013) Minireview: dopaminergic regulation of insulin secretion from the pancreatic islet. Mol. Endocrinol. 27, 1198–1207 23744894 10.1210/me.2013-1083 PMC3725340

[34] Julius D., Brake A., Blair L., Kunisawa R., and Thorner J. (1984) Isolation of the putative structural gene for the lysine-arginine-cleaving endopeptidase required for processing of yeast prepro-alpha-factor. Cell 37, 1075–1089 6430565 10.1016/0092-8674(84)90442-2

[35] Fricker L. D., Evans C. J., Esch F. S., and Herbert E. (1986) Cloning and sequence analysis of cdna for bovine carboxypeptidase-E. Nature 323, 461–464 3020433 10.1038/323461a0

[36] Hagedorn H. C., Jensen B. N., Krarup N. B., and Wodstrup I. (1936) Protamine insulinate. JAMA 106, 177–180 10.1001/jama.251.3.389 6361301

[37] Norrman M., Hubálek F., and Schluckebier G. (2007) Structural characterization of insulin NPH formulations. Eur. J. Pharm. Sci. 30, 414–423 17339105 10.1016/j.ejps.2007.01.003

[38] Smith G. D., Pangborn W. A., and Blessing R. H. (2001) Phase changes in T3R3f human insulin: temperature or pressure induced? Acta Crystallogr. D. 57, 1091–1100 11468392 10.1107/s0907444901007685

[39] Bentley G., Dodson G., and Lewitova A. (1978) Rhombohedral insulin crystal transformation. J. Mol. Biol. 126, 871–875 745246 10.1016/0022-2836(78)90026-8

[40] Wagner A., Diez J., Schulze-Briese C., and Schluckebier G. (2009) Crystal structure of ultralente: a microcrystalline insulin suspension. Proteins 74, 1018–1027 18767151 10.1002/prot.22213

[41] Steensgaard D. B., Schluckebier G., Strauss H. M., Norrman M., Thomsen J. K., Friderichsen A. V., Havelund S., and Jonassen I. (2013) Ligand-controlled assembly of hexamers, dihexamers, and linear multihexamer structures by the engineered acylated insulin degludec. Biochemistry 52, 295–309 23256685 10.1021/bi3008609

[42] Smeekens S. P., Avruch A. S., LaMendola J., Chan S. J., and Steiner D. F. (1991) Identification of a cdna-encoding a 2nd putative prohormone convertase related to Pc2 in Att20 cells and islets of Langerhans. Proc. Natl. Acad. Sci. U.S.A. 88, 340–344 1988934 10.1073/pnas.88.2.340 PMC50806

[43] Smeekens S. P., and Steiner D. F. (1990) Identification of a human insulinoma cdna-encoding a novel mammalian protein structurally related to the yeast dibasic processing protease Kex2. J. Biol. Chem. 265, 2997–3000 2154467

[44] Brader M. L., Kaarsholm N. C., Lee R. W., and Dunn M. F. (1991) Characterization of the R-state insulin hexamer and its derivatives: the hexamer is stabilized by heterotropic ligand-binding interactions. Biochemistry 30, 6636–6645 2065051 10.1021/bi00241a002

[45] Choi W. E., Brader M. L., Aguilar V., Kaarsholm N. C., and Dunn M. F. (1993) The allosteric transition of the insulin hexamer is modulated by homotropic and heterotropic interactions. Biochemistry 32, 11638–11645 8218231 10.1021/bi00094a021

[46] Huus K., Havelund S., Olsen H. B., Sigurskjold B. W., van de Weert M., and Frokjaer S. (2006) Ligand binding and thermostability of different allosteric states of the insulin zinc-hexamer. Biochemistry 45, 4014–4024 16548529 10.1021/bi0524520

[47] Lisi G. P., Png C. Y., and Wilcox D. E. (2014) Thermodynamic contributions to the stability of the insulin hexamer. Biochemistry 53, 3576–3584 24811232 10.1021/bi401678n

[48] Seydoux J., and Girardie L. (1974) Evidence for 2 receptor areas in brown adipose-tissue (Bat). Experientia 30, 683–683

[49] Arakawa T., Ejima D., Tsumoto K., Obeyama N., Tanaka Y., Kita Y., and Timasheff S. N. (2007) Suppression of protein interactions by arginine: a proposed mechanism of the arginine effects. Biophys. Chem. 127, 1–8 17257734 10.1016/j.bpc.2006.12.007

[50] Nuhu M. M., and Curtis R. (2015) Arginine dipeptides affect insulin aggregation in a pH- and ionic strength-dependent manner. Biotechnol. J. 10, 404–416 25611817 10.1002/biot.201400190

[51] Nicolson T. J., Bellomo E. A., Wijesekara N., Loder M. K., Baldwin J. M., Gyulkhandanyan A. V., Koshkin V., Tarasov A. I., Carzaniga R., Kronenberger K., Taneja T. K., da Silva Xavier G., Libert S., Froguel P., Scharfmann R., et al (2009) Insulin storage and glucose homeostasis in mice null for the granule zinc transporter ZnT8 and studies of the type 2 diabetes-associated variants. Diabetes 58, 2070–2083 19542200 10.2337/db09-0551 PMC2731533

[52] Fava E., Dehghany J., Ouwendijk J., Müller A., Niederlein A., Verkade P., Meyer-Hermann M., and Solimena M. (2012) Novel standards in the measurement of rat insulin granules combining electron microscopy, high-content image analysis and in silico modelling. Diabetologia 55, 1013–1023 22252472 10.1007/s00125-011-2438-4 PMC3296007

[53] Tsuboi T., and Rutter G. A. (2003) Multiple forms of “kiss-and-run” exocytosis revealed by evanescent wave microscopy. Curr. Biol. 13, 563–567 12676086 10.1016/s0960-9822(03)00176-3

[54] Conlon J. M. (2001) Evolution of the insulin molecule: insights into structure activity and phylogenetic relationships. Peptides 22, 1183–1193 11445250 10.1016/s0196-9781(01)00423-5

[55] Chami B., Steel A. J., De La Monte S. M., and Sutherland G. T. (2016) The rise and fall of insulin signaling in Alzheimer's disease. Metab. Brain. Dis. 31, 497–515 26883429 10.1007/s11011-016-9806-1

[56] Steen E., Terry B. M., Rivera E. J., Cannon J. L., Neely T. R., Tavares R., Xu X. J., Wands J. R., and de la Monte S. M. (2005) Impaired insulin and insulin-like growth factor expression and signaling mechanisms in Alzheimer's disease: is this type 3 diabetes? J. Alzheimers Dis. 7, 63–80 15750215 10.3233/jad-2005-7107

[57] Case D. A., Babin V., Berryman J. T., Betz R. M., Cai Q., Cerutti D. S., Cheatham T. E., Darden T. A., Duke R. E., Gohlke A. W., Goetz A. W., Gusarov S., Homeyer N., Janowski P., Kaus J., et al (2014) AMBER 14, University of California, San Francisco

[58] Salomon-Ferrer R., Götz A. W., Poole D., Le Grand S., and Walker R. C. (2013) Routine microsecond molecular dynamics simulations with AMBER on GPUs. 2. Explicit solvent particle mesh Ewald. J. Chem. Theory Comput. 9, 3878–3888 26592383 10.1021/ct400314y

[59] Duan Y., Wu C., Chowdhury S., Lee M. C., Xiong G., Zhang W., Yang R., Cieplak P., Luo R., Lee T., Caldwell J., Wang J., and Kollman P. (2003) A point-charge force field for molecular mechanics simulations of proteins based on condensed-phase quantum mechanical calculations. J. Comput. Chem. 24, 1999–2012 14531054 10.1002/jcc.10349

[60] Berendsen H. J. C., Grigera J. R., and Straatsma T. P. (1987) The missing term in effective pair potentials. J. Phys. Chem. 91, 6269–6271

[61] Berendsen H. J. C., Postma J. P. M., Vangunsteren W. F., Dinola A., and Haak J. R. (1984) Molecular dynamics with coupling to an external bath. J. Chem. Phys. 81, 3684–3690

[62] Essmann U., Perera L., Berkowitz M. L., Darden T., Lee H., and Pedersen L. G. (1995) A smooth particle mesh Ewald method. J. Chem. Phys. 103, 8577–8593

[63] Miyamoto S., and Kollman P. A. (1992) Settle: an analytical version of the shake and rattle algorithm for rigid water models. J. Comput. Chem. 13, 952–962

[64] Hutton J. C. (1982) The internal pH and membrane potential of the insulin-secretory granule. Biochem. J. 204, 171–178 6126183 10.1042/bj2040171 PMC1158329

[65] Ishikawa T., Chatake T., Morimoto Y., Maeda M., Kurihara K., Tanaka I., and Niimura N. (2008) An abnormal pK (a) value of internal histidine of the insulin molecule revealed by neutron crystallographic analysis. Biochem. Biophys. Res. Commun. 376, 32–35 18725203 10.1016/j.bbrc.2008.08.071

[66] Bryant C., Spencer D. B., Miller A., Bakaysa D. L., McCune K. S., Maple S. R., Pekar A. H., and Brems D. N. (1993) Acid stabilization of insulin. Biochemistry 32, 8075–8082 8394123 10.1021/bi00083a004

[67] Chang X., Jorgensen A. M., Bardrum P., and Led J. J. (1997) Solution structures of the R-6 human insulin hexamer. Biochemistry 36, 9409–9422 9235985 10.1021/bi9631069

[68] Wang J., Wang W., Kollman P. A., and Case D. A. (2006) Automatic atom type and bond type perception in molecular mechanical calculations. J. Mol. Graph. Model 25, 247–260 16458552 10.1016/j.jmgm.2005.12.005

[69] Frisch M. J. T., Schlegel G. W. H. B., Scuseria G. E., Robb M. A., Cheeseman J. R., Scalmani G., Barone V., Mennucci B., Petersson G. A., Nakatsuji H., Caricato M., Li X., Hratchian, et al (2009) Gaussian 09, Revision A.1. Wallingford, CT

[70] Leontyev I., and Stuchebrukhov A. (2011) Accounting for electronic polarization in non-polarizable force fields. Phys. Chem. Chem. Phys. 13, 2613–2626 21212894 10.1039/c0cp01971b

[71] Leontyev I. V., and Stuchebrukhov A. A. (2012) Polarizable mean-field model of water for biological simulations with AMBER and CHARMM force fields. J. Chem. Theory Comput. 8, 3207–3216 25580096 10.1021/ct300011h PMC4285689

[72] Leontyev I. V., and Stuchebrukhov A. A. (2014) Polarizable molecular interactions in condensed phase and their equivalent nonpolarizable models. J. Chem. Phys. 141, 014103 10.1063/1.4884276 PMC4106032 25005273

[73] Kohagen M., Mason P. E., and Jungwirth P. (2016) Accounting for electronic polarization effects in aqueous sodium chloride via molecular dynamics aided by neutron scattering. J. Phys. Chem. B 120, 1454–1460 26172524 10.1021/acs.jpcb.5b05221

[74] Winter G. (2010) xia2: an expert system for macromolecular crystallography data reduction. J. Appl. Crystallogr. 43, 186–190

[75] Emsley P., and Cowtan K. (2004) Coot: model-building tools for molecular graphics. Acta Crystallogr. D 60, 2126–2132 15572765 10.1107/S0907444904019158

[76] Collaborative Computational Project, Number 4 (1994) The Ccp4 suite: programs for protein crystallography. Acta Crystallogr. D 50, 760–763 15299374 10.1107/S0907444994003112

[77] Vagin A., and Teplyakov A. (1997) MOLREP: an automated program for molecular replacement. J. Appl. Crystallogr. 30, 1022–1025

[78] Smith G. D., Pangborn W. A., and Blessing R. H. (2003) The structure of T-6 human insulin at 1.0 angstrom resolution. Acta Crystallogr. D Biol. Crystallogr. 59, 474–482 12595704 10.1107/s0907444902023685

[79] Murshudov G. N., Vagin A. A., and Dodson E. J. (1997) Refinement of macromolecular structures by the maximum-likelihood method. Acta Crystallogr. D. 53, 240–255 15299926 10.1107/S0907444996012255

[80] McNicholas S., Potterton E., Wilson K. S., and Noble M. E. (2011) Presenting your structures: the CCP4mg molecular-graphics software. Acta Crystallogr. D. 67, 386–394 21460457 10.1107/S0907444911007281 PMC3069754

[81] Huang S. T., Choi W. E., Bloom C., Leuenberger M., and Dunn M. F. (1997) Carboxylate ions are strong allosteric ligands for the His^B10^ sites of the R-state insulin hexamer. Biochemistry 36, 9878–9888 9245420 10.1021/bi9701639

[82] Bloom C. R., Wu N., Dunn A., Kaarsholm N. C., and Dunn M. F. (1998) Comparison of the allosteric properties of the Co(II)- and Zn(II)-substituted insulin hexamers. Biochemistry 37, 10937–10944 9692986 10.1021/bi980071z

[83] Cornell W. D., Cieplak P., Bayly C. I., and Kollmann P. A. (1993) Application of RESP charges to calculate conformational energies, hydrogen bond energies, and free energies of solvation. J. Am. Chem. Soc. 115, 9620–9631

[84] Reed A. E., Weinstock R. B, and Weinhold F. (1985) Natural population analysis. J. Chem. Phys. 83, 735–746

[85] Becke A. D. (1993) Density-functional thermochemistry. III. The role of exact exchange. J. Chem. Phys. 98, 5648–5652

[86] Lee C., Yang W., and Parr R. G. (1988) Development of the Colle-Salvetti correlation-energy formula into a functional of the electron density. Phys. Rev. B 37, 785–789 10.1103/physrevb.37.785 9944570

