# Supplementary material for: Computational and structural evidence for neurotransmitter-mediated modulation of the oligomeric states of human insulin in storage granules
Source: J Biol Chem. 2017 Mar 27;292(20):8342–55. doi: 10.1074/jbc.M117.775924 (PMC5437240; doi:10.1074/jbc.M117.775924)
Supplement: Supplemental Data [file supp_292_20_8342__index.html]

Computational and Structural Evidence for Neurotransmitter-mediated Modulation of the Oligomeric States of Human Insulin in Storage Granules — Computational and structural evidence for neurotransmitter-mediated modulation of the oligomeric states of human insulin in storage granules — Structural forms of storage insulin in pancreas — Supplemental Data 

# Computational and structural evidence for neurotransmitter-mediated modulation of the oligomeric states of human insulin in storage granules

## Supplemental Data

- Supplementary Information (.pdf, 7.1 MB) - Supplementary Information containing details of the X-ray Data, Molecular Dynamics calculation and spectroscopic studies
