# Supplementary material for: Computational and structural evidence for neurotransmitter-mediated modulation of the oligomeric states of human insulin in storage granules
Source: J Biol Chem. 2017 Mar 27;292(20):8342–55. doi: 10.1074/jbc.M117.775924 (PMC5437240; doi:10.1074/jbc.M117.775924)
Supplement: Supplemental Data [file 10.1074_M117.775924_jbc.M117.775924-1.pdf]

Supplementary Information:

Computational and Structural Evidence for Neurotransmitter-mediated Modulation of the Oligomeric States of Human Insulin in Storage Granules

Vladimír Palivec[a], Cristina M. Viola[b], Mateusz Kozak[b≠], Timothy R. Ganderton[b], , Květoslava Křížková,[a] Johan P. Turkenburg[b], , Petra Halušková,[a] Lenka Žáková[a], Jiří Jiráček[a*], Pavel Jungwirth[a*], Andrzej M. Brzozowski[b*]

(i) Table S1. X-ray data collection and refinement statistics.

	InsSerT_3R_3	InsSerArgf1-T_3R_3	InsSerArgf2-T_3R_3
**PDB Code**	5MAM	5MT3	5MT9
**Data collection**			
Beamline	DLS, I03	DLS, I04	DLS, I04
Wavelength (Å)	0.97631	0.97949	0.97949
Space group	*H*3	*H*3	*H*3
Cell dimensions			
a, *b*, *c* (Å)	158.96 158.96 76.53	158.64 158.64 76.10	159.31 159.31 76.50
α, β, γ (°)	90.0 90.0 120.0	90.0 90.0 120.0	90.0 90.0 120.0
Resolution (Å)	51.2-2.20(2.26-2.20)	42.89-2.02(2.07-.02)	46.0-1.88(1.93-1.88)
N^o. mol/AU	16	16	16
R_{sym}	0.082(0.919)	0.059(0.689)	0.049(1.086)
R_{pim}	0.042(0.657)	0.045(0.521)	0.031(0.694)
$<I / \sigma(I)>$	13.3(2.2)	13.6(2.6)	15.1(1.6)
Completeness (%)	98.4(94.2)	98.3(97.7)	98.3(89.7)
Redundancy	4.8(4.2)	4.9(4.6)	5.0(4.4)
**Refinement**			
Resolution (Å)	51.2-2.20	42.89-2.02	37.34 - 2.15
No. of reflections	34232	46012	57913
R_{work} / R_{free}	0.206/0.28	0.24/0.31	0.207/0.269
No. atoms	6161	6162	6223
Protein	5705	5652	5666
Ligand	182	204	256
Ions	16	14	16
Water	258	292	285
B-factors (Å^2)			
Wilson	36.5	31.9	31.4
Protein	46.8	43.0	41.8
Ligand	52.3	51.6	51.8
Ions	40.4	53.5	34.7
Water	49.8	46.9	46.0
R.m.s. deviations			
Bond lengths (Å)	0.014	0.014	0.017
Bond angles (°)	1.666	1.692	1.851
**Ramachandran (%)**			
Preferred/Allowed/ Outliers	96.9/2.7/0.4	97.2/2.1/0.7	98.8/0.9/0.3

*Values in parentheses are for highest-resolution shell.

(ii) Crystallization conditions.

For all complexes Zn-free human insulin (gift from Novo Nordisk) was dissolved in 20 mM HCl at 5 mg/mL. Hanging drop method was used for all setups with Linbro dishes, at the 1:1 protein:well volumes ratio. The Zn^{2+} concentration varied from 0.4 mM to 8 mM, the serotonin from 10mM to 40 mM, and arginine from 20mM to 100mM. The compositions of the well solutions for the successful crystallizations of each complex are given below. All crystallizations were carried out at 293K.

InsSerT$_3$R$_3$: 5 mM ZnAcetate, 35 mM NaCitrate, 1.1 M NaCl, 0.3M Tris pH 7.5, 40 mM serotonin.

InsSerArgf1-T$_3$R$_3$ and ***InsSerArgf2-T$_3$R$_3$:*** 5 mM ZnAcetate, 35 mM NaCitrate, 1.1 M NaCl, 0.3M Tris pH 7.5, 40 mM serotonin, 100 mM arginine.

Insulin:neurotransmitters:Arg crystallizations were also carried out in the presence of Ca^{2+} ($CaCl_2$) at 5, 10 and 20 mM concentrations. Resulted structures did not differ from the hexamers obtained in the absence of Ca^{2+} hence they are not reported here being redundant.

(iii) Partial charges for molecular dynamics simulation

In Table S2 and Figure S5, we provide the list partial atomic charges on the phenolic ligands.

(iv) Free energy calculations

Binding site I – the phenolic pocket

The binding free energy differences $\Delta\Delta G$ between phenol, dopamine, and serotonin were calculated using the thermodynamic integration method, combined with calculation of absolute free energy of binding of the phenol molecule using double annihilation method (1). Composition of the systems was as follows – one insulin R_6(phenol)$_5$ hexamer, 9 000 SPC/E water molecules with Na^+/Cl^- ions added to ensure overall electroneutrality with no excess of salt, and one phenolic ligand (phenol, dopamine, or serotonin) inside the last free phenolic pocket. Differences in free energy of binding $\Delta\Delta G_{1\rightarrow2}$ between phenol (PHN), dopamine (DPN), and serotonin (SEN) were calculated. For these types of calculations, a complete thermodynamic cycle shown in Figure S6 was used (example calculation for $\Delta\Delta G_{\mathrm{PHN}\rightarrow\mathrm{SEN}}$).

In Figure S6, ΔG_{b1} represents free energy of binding of a ligand 1 to the insulin while ΔG_{d2} represents free energy of dissociation of a ligand 2 from the insulin. $\Delta\Delta G_{1\rightarrow2}$ reflects the difference in free energies of binding between these two ligands. As this is a complete thermodynamic cycle, this free energy equals to

$$\Delta G_{1\rightarrow2} = -\Delta G_{b1} - \Delta G_{d2}, \qquad \text{SI.1}$$
$$\Delta G_{1\rightarrow2} = \Delta G_1 + \Delta G_2 + \Delta G_3 + \Delta G_4 + \Delta G_5 + \Delta G_6 + \Delta G_{ron} + \Delta G_{roff}. \qquad \text{SI.2}$$

Each subsequent simulation was performed using linear scaling between the initial and final potentials with lambda windows 0, 0.1, up to 1.0, resulting in 11 windows. The only exception were simulations where the van der Waals parameters were changed with a lambda window of 0.05. All simulations were performed with a simulation step of 2 fs for a total simulation time 5 ns (at first) with preceding 1.2 ns equilibration. Altogether, a single calculation of binding free energy difference $\Delta\Delta G_{1\rightarrow2}$ consisted of 108 subsequent simulations. In order to be able to reasonably estimate an error in these calculations, every calculation was performed in both

directions (forward and backwards mutations) and multiple times. This led to 6 separate mutations with the following differences in binding free energies $\Delta\Delta G_{PHN\rightarrow DPN}$, $\Delta\Delta G_{DPN\rightarrow PHN}$, $\Delta\Delta G_{PHN\rightarrow SEN}$, $\Delta\Delta G_{SEN\rightarrow PHN}$, $\Delta\Delta G_{DPN\rightarrow SEN}$, and $\Delta\Delta G_{SEN\rightarrow DPN}$.

To obtain the absolute free energy of a phenol molecule binding to a phenolic pocket ΔG_{PHN}, a thermodynamic cycle shown in Figure S7 was used. The computational protocol was as follows. Each subsequent simulation was performed using linear scaling between the initial and final potentials. The equilibrium values were taken from a non-restrained molecular dynamics simulation. The process of calculating the free energy difference of restraining the phenol (using restraints proposed by Boresch (1))was broken into 12 windows. Each window was equilibrated for 2 ns and then the data were gathered for 5 ns. The second step was turning the electrical charges of the phenol in the binding pocket off (ΔG_8), while the restraints are on. This was done in 11 windows, each equilibrated for 2 ns, followed by 30 ns data acquisition. The next step involved full decoupling of the phenol while the proposed restrains stayed on and the electrical charged turned off (ΔG_9). This calculation was divided into 33 windows, each equilibrated for 2 ns, followed by 50 ns of data collection. The next step, the transition from the bound to the unbound state (bulk solution) has a zero free energy difference $\Delta G_{10} = 0$ as the ligand gets fully decoupled from its environment. The following step is releasing the restraints from the phenol (ΔG_{11}) with correction to a standard concentration of 1 M. After restraints got released, the only remaining steps are to turn on the van der Waals interactions (ΔG_{12}) and electrical charges of the phenol (ΔG_{13}) in the bulk solution. The van der Waals interactions were turned on in 21 windows, each equilibrated for 2 ns, and followed by 10 ns of data collection. The electrostatic interactions were turned on in 11 windows, each equilibrated for 2 ns, and followed by 10 ns of data collection.

Summing all the terms, we obtain the standard free energy difference of decoupling the phenol from the phenolic pocket ΔG_{PHN}

$$\Delta G_{PHN} = -(\Delta G_7 + \Delta G_8 + \Delta G_9 + \Delta G_{11} + \Delta G_{12} + \Delta G_{13}). \qquad \text{SI.3}$$

However, due to the symmetry of insulin R_6 hexamer, there is also an additional contribution to the free energy of binding. There are 6 equivalent binding sites for a phenol molecule. To account for this degeneracy entropy effect, the final free energy of binding has to be adjusted by a factor of

$$\Delta G_{symm\_i} = -RT\ln(i), \qquad \text{SI.4}$$

where the value of i depends on the number of the remaining free binding sites for the phenol molecule. For R_6 insulin hexamer without any phenol bound, i equals 6 hence the contribution is the highest. This number goes to zero as the phenolic pockets get fully occupied by phenol molecules. Table S3 summarizes the values ΔG_{symm_i}.

By combining the above two approaches with an average entropy value $\langle\Delta G_{symm}\rangle$ one gets the absolute standard free energies of binding of phenol, serotonin, and dopamine to a phenolic pocket of the insulin R_6 hexamer:

$$\Delta G^{\circ}_{PHN} = \Delta G_{PHN} + \langle\Delta G_{symm}\rangle, \qquad \text{SI.5}$$

$$\Delta G^{\circ}_{DPN} = \Delta G^{\circ}_{PHN} + \Delta\Delta G_{PHN\rightarrow DPN}, \qquad \text{SI.6}$$

$$\Delta G^{\circ}_{SEN} = \Delta G^{\circ}_{PHN} + \Delta\Delta G_{PHN\rightarrow SEN}. \qquad \text{SI.7}$$

Binding site III

As the three equivalent binding sites III are located on the surface of the insulin hexamer, umbrella sampling turns out to be a suitable method for obtaining binding free energies. As a reaction coordinate, the distance from the center of mass of the insulin hexamer to center of mass of heavy atoms of the phenolic ligand was used. The calculations were performed using 21 evenly spaced windows. This was followed by production runs in each window of 50 ns (phenol), 80ns (dopamine), or 100 ns (serotonin). Free energy profiles were then constructed using the WHAM procedure

As our reaction coordinate is expressed in spherical coordinates, one has to correct the results by a Jacobian factor of$-\mathbf{R}T\ln(4\pi \boldsymbol{r}^2)$, where **R** is a molar gas constant, *T* stands for the thermodynamic temperature, and ***r*** is the distance used in each of the umbrella window. This correction is already accounted for in presented potentials of mean force $W(r)$. Using a square well potential approximation the dissociation constant K_d can be expressed as follows

$K_d = V_b\exp(W_0/\mathbf{R}T)$,

SI.8where V_b stands for the volume occupied by the ligand when bound to the protein, and W_0 represents the depth of the potential well

$W_0 = W(r_{\rightarrow}r_{flat}) - W(r_{\rightarrow}r_{min})$,

SI.9where r_{min} denotes minimum in the potential of the mean force calculated by the umbrella sampling, and r_{flat} represents the distance where the potential gets flat (bulk solution). The dissociation constant K_d in the context of simple protein-ligand binding is then related to the standard free energy of binding ΔG°_b by

$\Delta G^{\circ}_b = \mathbf{R}T\ln(C^{\circ} K_d)$,

SI.10where C° stands here for a standard concentration. The inverse of the standard concentration can be interpreted as the volume V° occupied by a single molecule at standard concentration 1 M. Finally, Table S4 presents entropy corrections to the free energy of binding of phenolic ligand to binding site III because of the symmetry of the insulin hexamer with three equivalent binding sites.

The resulting standard free energy of binding ΔG°_b,which is calculated by the umbrella sampling method and corrected for symmetry, is then given as

$\Delta G^{\circ}_b = W_0 + \mathbf{R}T\ln(V_b/V_0) + \langle \Delta G_{symm} \rangle$, SI.11

(v) Table S2. List of partial atomic charges of phenolic ligands used in this work. Charges were calculated by a standard HF/6-31G* method in vacuum using RESP method.

	Column2	Column3	Column4	Column5	Column6	Column7	Column8	Column9	Column10	Column11	Column12
[name]	C1	H1	C2	H2	C3	H3	C4	O	OH		
phenol	-0.143019	0.136313	-0.172073	0.157855	-0.194232	0.152367	0.264153	-0.521170	0.375888		
[name]	O1	OH1	O2	OH2	N	HN	C1	H1	C2	H2	
dopamine	-0.593381	0.478962	-0.616674	0.482245	-0.252754	0.289534	-0.344643	0.195479	-0.247315	0.225314	
[name]	C3	C4	C5	H5	C6	C7	H7	C8	H8		
dopamine	0.362755	0.208789	-0.321489	0.182811	0.082771	-0.141354	0.111003	-0.020626	0.114250		
[name]	C1	H1	N1	HN1	C2	C3	H3	C4	H4	C5	O
serotonin	-0.173890	0.209065	-0.329917	0.362564	0.115484	-0.206182	0.189443	-0.256801	0.164268	0.336480	-0.629844
[name]	HO	C6	H6	C7	C8	C9	H9	C10	H10	N2	HN2
serotonin	0.466462	-0.299910	0.198943	-0.007419	-0.033277	-0.146931	0.088605	0.037951	0.094844	0.256820	0.297811

(vi) Table S3. Entropy contribution to the free energy of binding of a phenol to the phenolic pockets according to how many phenolic pockets are unoccupied (ΔG_{symm_i}).

i	6	5	4	3	2	1	$\frac{\langle \Delta G_{symm} \rangle}{kcal/mol}$
$\frac{\Delta G_{symm\_i}}{kcal/mol}$	-1.07	-0.96	-0.83	-0.65	-0.41	0.00	-0.65

(vii) Table S4. Entropy contribution to the free energy of binding of a phenolic ligand to binding site III according to how many pockets are unoccupied ($\Delta G_{\mathrm{symm\ i}}$).

i	3	2	1	$\frac{\langle \Delta G_{symm} \rangle}{kcal/mol}$
$\frac{\Delta G_{symm\ i}}{kcal/mol}$	-0.65	-0.41	0.00	-0.36

(viii) Figure S1. Electrostatic surface representation of the main forms of insulin hexamers (top views as in Figure 1). The increasing structural occlusion of the Zn-neighborhood sphere in the TR transition is shown.

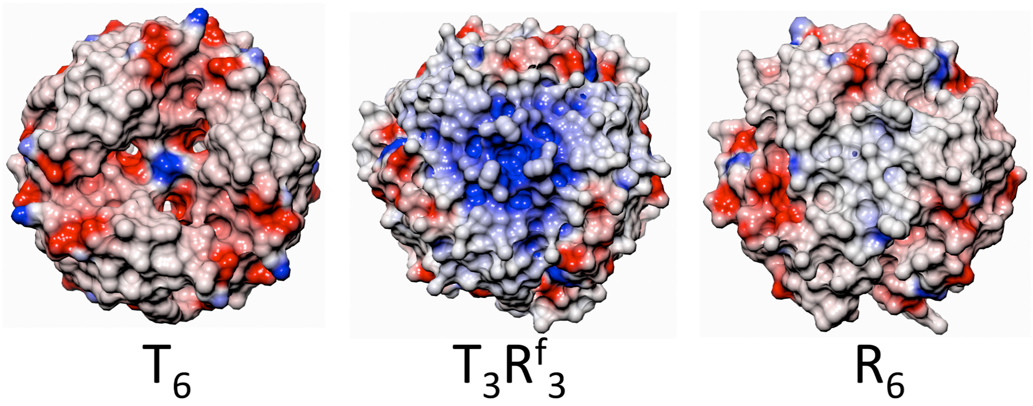

(ix) Figure S2. Scatter plot representation of the data shown in Table 3. **A.** K_d values. **B.** B_{max} values. **C.** Hill coefficient (h) values.

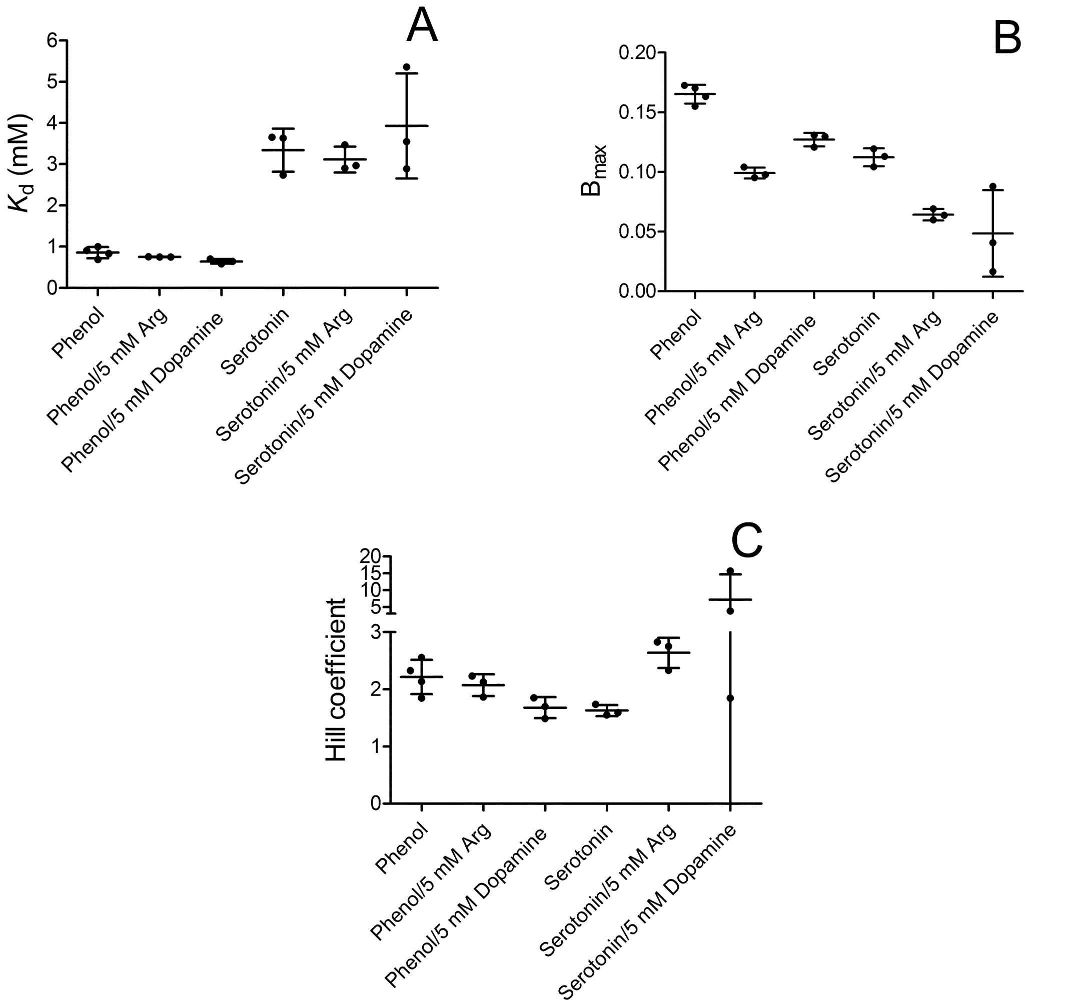

(x) Figure S3. An example of the FWPHWT electron density map (blue mesh) for the serotonin in site I, contoured at 1σ level. Labelling as for Figure 8 (top).

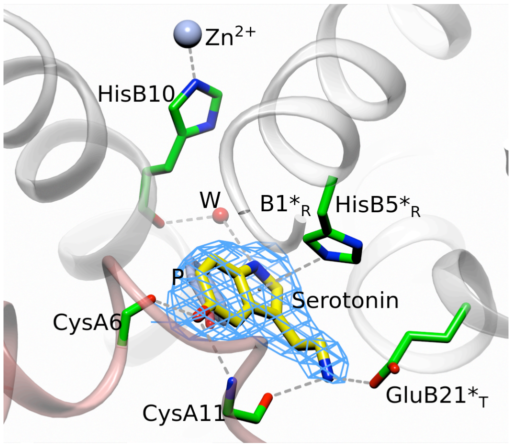

(xi) Figure S4. An example of the FWPHWT electron density map (blue mesh) for the arginine binding sites in insulin InsSerArgT_3R_3 hexamer, contoured at 1σ level. Labelling as for Figure 10. Some interactions showed in Figure 10 are omitted for image clarity.

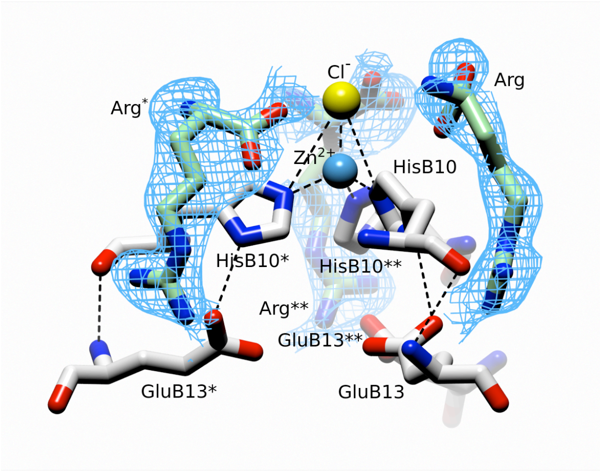

(xii) Figure S5. Labelling of the phenolic ligands (left – phenol, middle – dopamine, right – serotonin). Partial atomic charges are listed in Table S1.

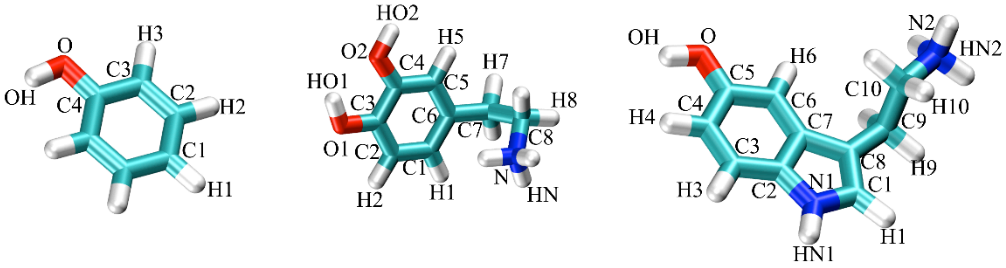

(xiii) Figure S6. A practical example of a complete thermodynamic cycle used to calculate the differences in the free energies of binding $\Delta\Delta G_{1\rightarrow2}$ between different phenolic ligands to phenolic pocket of insulin R_6 hexamer. A thermodynamic cycle to calculate $\Delta\Delta G_{PHN\rightarrow SEN}$ is shown here. The restraints are indicated by a red circle; the grey ligand indicates that the electrostatic interactions of a ligand are turned off. ΔG_{b1} represents free energy of binding of phenol to the phenolic pocket whereas ΔG_{d2} represents free energy of dissociation of serotonin from the phenolic pocket. ΔG_{ro} represents free energy of restraining phenol to a certain position inside the phenolic pocket. ΔG_1 represents gradual turning off electrical charges of phenol inside the phenolic pocket while keeping proposed restraints on. ΔG_2 represents mutation of a restrained phenol to a restrained serotonin inside the phenolic pocket while all charges are turned off. ΔG_3 represents free energy of turning on the electrical charges of the serotonin while the restrains are on. ΔG_{roff} represents free energy of releasing restraints on the serotonin inside the phenolic pocket. ΔG_4 stands for free energy of turning off electrical charges of the serotonin in the bulk solution. ΔG_5 represents free energy of mutating serotonin to phenol while all electrical charges are off in the bulk solution. ΔG_6 represents free energy of turning on electrical charges of phenol in the bulk solution.

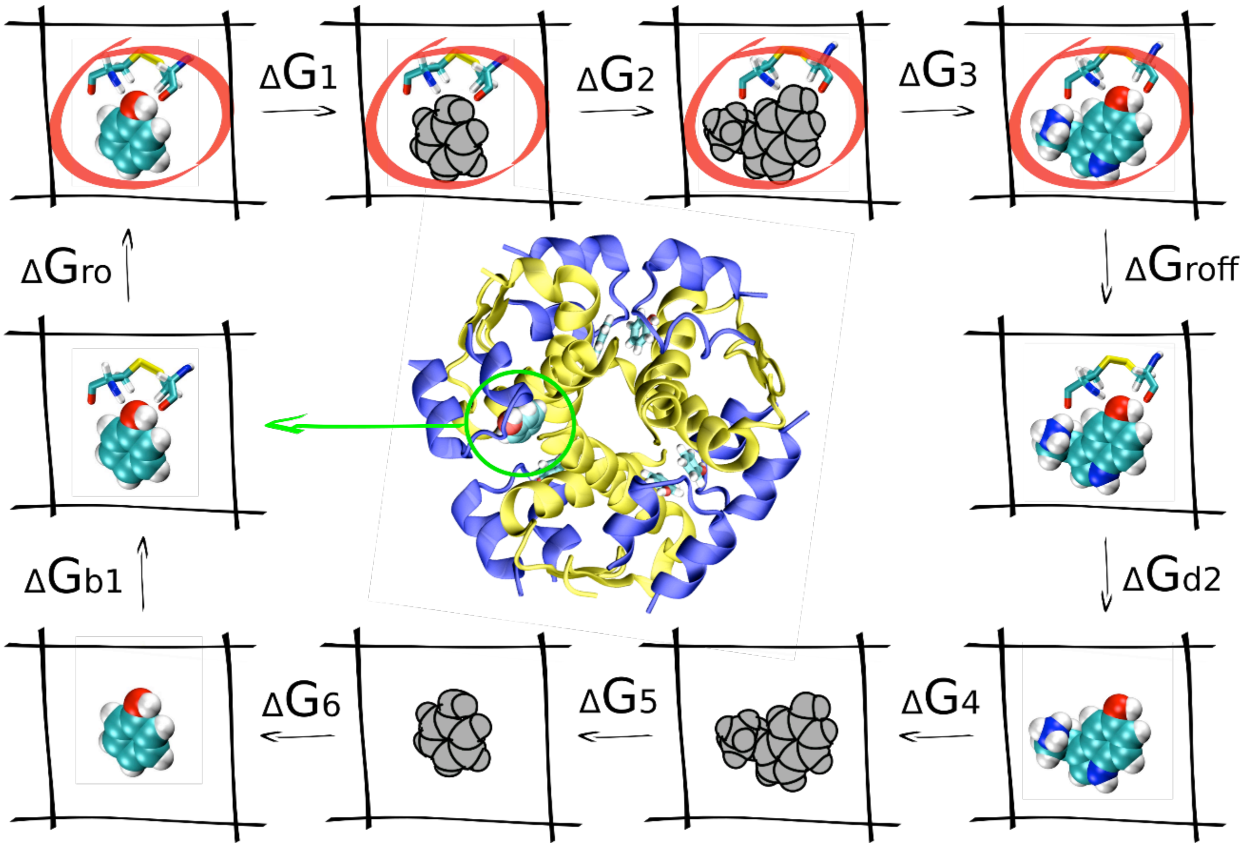

(xiv) Figure S7. A complete thermodynamic cycle used to calculate the absolute free energy of binding of a phenol molecule to a phenolic pocket of insulin R_6 hexamer ΔG_{PHN}. The restraints are depicted by a red circle; grey ligand means that the electrostatic interactions are turned off; fully transparent ligand means that the ligand is fully decoupled from its environment. ΔG_7 represents free energy of restraining phenol to a certain position inside the phenolic pocket. ΔG_8 represents free energy of turning off electrical charges of phenol inside the phenolic pocket while keeping the proposed restraints on. ΔG_9 stands for the free energy of decoupling restrained phenol from the phenolic pocket while all charges stay turned off. ΔG_{10} stands for the free energy of transferring decoupled phenol from phenolic pocked to the bulk water environment. As the phenol does not interact with the protein at all, the energy of bound and unbound phenol is the same. Hence the free energy difference between these two states is zero. ΔG_{11} represents free energy of releasing the proposed restrains from phenol, which is now situated in the bulk solution. ΔG_{12} represents the free energy of turning on the van der Waals interactions of phenol in the bulk solution. ΔG_{13} stands for the free energy of turning on electrical charges of phenol in the bulk solution.

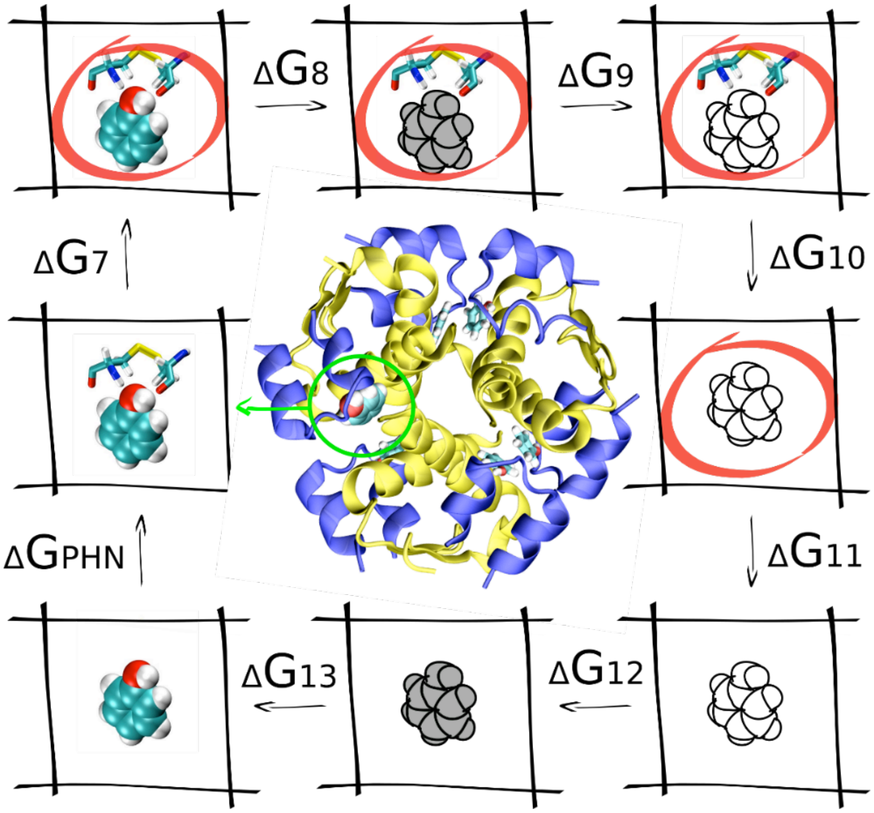

1. Boresch, S., Tettinger, F., Leitgeb, M., and Karplus, M. (2003) Absolute binding free energies: A quantitative approach for their calculation. *J. Phys. Chem. B* **107**, 9535-9551
